# The Rat microRNA body atlas; Evaluation of the microRNA content of rat organs through deep sequencing and characterization of pancreas enriched miRNAs as biomarkers of pancreatic toxicity in the rat and dog

**DOI:** 10.1186/s12864-016-2956-z

**Published:** 2016-08-30

**Authors:** Aaron Smith, John Calley, Sachin Mathur, Hui-Rong Qian, Han Wu, Mark Farmen, Florian Caiment, Pierre R. Bushel, Jianying Li, Craig Fisher, Patrick Kirby, Erik Koenig, David G. Hall, David E Watson

**Affiliations:** 1 Department of Investigative Toxicology, Non Clinical Safety Assessment and Pathology, Lilly Research Laboratories, Lilly Corporate Center, Indianapolis, 46285 IN USA; 2 Department of TTX Bioinformatics, Lilly Research Laboratories, Lilly Corporate Center, Indianapolis, 46285 IN USA; 3 Department of Discovery and Development Statistics, Lilly Research Laboratories, Lilly Corporate Center, Indianapolis, 46285 IN USA; 4 Department of Toxicogenomics, Maastricht University, Universiteitsingel, Maastricht, The Netherlands; 5 National Institute of Environmental Health Sciences, Biostatistics Branch, Durham, NC USA; 6 Kelly Government Solutions, Research Triangle Park, Durham, NC 27709 USA; 7 Drug Safety Evaluation, Takeda Pharmaceuticals International Company, Deerfield, USA; 8 Molecular Pathology, Takeda Pharmaceuticals International Company, Deerfield, USA; 9 Department of Investigative Pathology, Lilly Research Laboratories, Lilly Corporate Center, Indianapolis, 46285 IN USA

**Keywords:** microRNA, Biomarkers, Deep sequencing, Tissue specific, Tissue enriched, Pancreatic toxicity

## Abstract

**Background:**

MicroRNAs (miRNA) are ~19–25 nucleotide long RNA molecules that fine tune gene expression through the inhibition of translation or degradation of the mRNA through incorporation into the RNA induced silencing complex (RISC). MicroRNAs are stable in the serum and plasma, are detectable in a wide variety of body fluids, are conserved across veterinary species and humans and are expressed in a tissue specific manner. They can be detected at low concentrations in circulation in animals and humans, generating interest in the utilization of miRNAs as serum and/or plasma based biomarkers of tissue injury. MicroRNA tissue profiling in rodents has been published, but sample an insufficient number of organs of toxicologic interest using microarray or qPCR technologies for miRNA detection. Here we impart an improved rat microRNA body atlas consisting of 21 and 23 tissues of toxicologic interest from male and female Sprague Dawley rats respectively, using Illumina miRNA sequencing. Several of the authors created a dog miRNA body atlas and we collaborated to test miRNAs conserved in rat and dog pancreas in caerulein toxicity studies utilizing both species.

**Results:**

A rich data set is presented that more robustly defines the tissue specificity and enrichment profiles of previously published and undiscovered rat miRNAs. We generated 1,927 sequences that mapped to mature miRNAs in rat, mouse and human from miRBase and discovered an additional 1,162 rat miRNAs as compared to the current number of rat miRNAs in miRBase version 21. Tissue specific and enriched miRNAs were identified and a subset of these miRNAs were validated by qPCR for tissue specificity or enrichment. As an example of the power of this approach, we have conducted rat and dog pancreas toxicity studies and examined the levels of some tissue specific and enriched miRNAs conserved between rat and dog in the serum of each species. The studies demonstrate that conserved tissue specific/enriched miRs-216a-5p, 375-3p, 148a-3p, 216b-5p and 141-3p are candidate biomarkers of pancreatic injury in the rat and dog.

**Conclusions:**

A microRNA body atlas for rat and dog was useful in identifying new candidate miRNA biomarkers of organ toxicity in 2 toxicologically relevant species.

**Electronic supplementary material:**

The online version of this article (doi:10.1186/s12864-016-2956-z) contains supplementary material, which is available to authorized users.

## Background

The drug discovery process is a long and expensive endeavor with many experimental molecules failing in late stage in vivo toxicology studies. The high attrition rate of drugs in late stage clinical trials lead to the call for investigation of the utilization of species translatable biomarkers to select safer compounds early in the drug development process [1]. In order to better understand toxicities in preclinical and clinical settings and to speed the development of new therapies, body fluid based biomarkers of organ damage have been sought to both identify and monitor toxicities. Serum or plasma based biomarkers capable of monitoring tissues affected by drug toxicity could allow for the detection of tissue injury from simple blood draws, thus permitting larger numbers of compounds to be tested more rapidly in studies that are less expensive and use fewer animals compared to traditional means of toxicity evaluation. The properties of an ideal biomarker include tissue specificity, a strong correlation to organ dysfunction or toxicity, a strong association to histopathologic changes, robust sensitivity, predictability, accessibility through a noninvasive method, readily-available rapid and inexpensive assays for quantitative measurement and direct translatability across preclinical species and human.

Serum based biomarkers of organ dysfunction have been useful in preclinical and clinical settings. Serum AST (aspartate aminotransferase) and ALT (alanine aminotransferase) were discovered in 1957 [2] and have been used in combination with total bilirubin, gamma-glutamyl transferase (GGT), alkaline phosphatase (ALP), albumin and bile acids as markers of liver injury to de-risk compounds in preclinical drug development [3, 4] as well as being utilized for monitoring liver toxicity in clinical drug development [5]. Additional markers of hepatotoxicity are currently under investigation [6]. Cardiac troponin I is a sensitive and specific serum based biomarker of cardiac injury in humans and mice and has been employed to mitigate cardiac toxicity in mouse toxicology studies with novel kinase inhibitors [7]. Urine based biomarkers for evaluation of renal toxicity include BUN (blood urea nitrogen), creatinine, urinary albumin, KIM-1 (kidney injury molecule-1), NGAL (neutrophil gelatinase associated lipocalin) and cystatin C [8]. Amylase and lipase have been used as markers for pancreatitis, but their diagnostic capability is limited by sensitivity and specificity [9]. These preclinical and clinical biomarkers are carefully reviewed in Dieterle, 2009 [8]. Despite the success with protein based biomarkers to date, gaps in our ability to detect toxicities in a number of organs prevent the widespread utilization of biomarkers in toxicology studies. Current gaps in the minimally-invasive biomarker portfolio include gastrointestinal injury, CNS (central nervous system) injury, reproductive toxicity and specific markers of glomerular and liver injury. Clinical chemistry parameters offer important information regarding the health of a given organ but can be limited by lack of specificity and sensitivity as in the cases of ALT and AST for liver injury and amylase and lipase for injury in the exocrine pancreas [8]. While protein based biomarkers provide useful information, they can be limited in their translation across species and to the clinical setting due to species differences in the biomarkers and the need for species specific immunoassay reagents.

Traditional pre-clinical methods for assessing the potential toxicity of a compound include clinical chemistry, histopathology, hematology, physiological measurements and clinical observations, but the concordance rate to human toxicity was reported by Olsen et al. to be 71 % leaving room for significant improvement in preclinical toxicology studies [10]. Unfortunately, the concordance of clinical chemistry or other biomarkers was not directly compared to establish concordance between preclinical and clinical studies; Several groups advised more use of biomarkers in preclinical and clinical studies [10]. In a subsequent study, adverse drug reactions in each target organ from compounds approved in Japan from 2001–2010 were found to be 48 % concordant between preclinical toxicology studies and humans while changes in laboratory parameters displayed a 30-70 % correlation [11]. It is evident that additional endpoints indicative of organ toxicities that are translatable and/or predictive from preclinical species to human are necessary to improve the safety of compounds in preclinical drug development and clinical trials. With the end goal of identifying tissue specific, species translatable blood based biomarkers of tissue injury, we set out to characterize the rat microRNA expression profile in various organs and create a rat microRNA body atlas to identify specific and enriched miRNAs in individual tissues.

MicroRNAs are 21–25 nucleotide long RNA molecules whose cellular function appears to be to inhibit translation or cause degradation of their mRNA target or targets [12]. Some miRNAs are expressed in high abundance in a tissue specific manner, are resistant to degradation in the serum, are well conserved in preclinical species including mouse, rat, macaque and human, and are detectable in a wide variety of biologic fluids [13–21]. In addition, sensitive, specific and readily available qPCR based assays exist to measure miRNAs making them attractive candidates for serum based biomarkers for organ injury. Perhaps the most well characterized miRNA for use as a toxicity biomarker is the liver enriched miR-122-5p. This miRNA was increased in the serum of rats that were given the liver toxicant trichlorobromomethane, but not increased in the serum of rats administered the skeletal muscle toxicants 2,3,5,6 tetramethyl-p-phenylenediamine (TMPD) and an HMG-CoA reductase inhibitor [22]. AST and ALT were increased when animals were given liver or skeletal muscle toxicants, demonstrating that miR-122 was indicative of liver injury only and not muscle injury, thus displaying an advantage in specificity over ALT and AST. Mice treated with acetaminophen displayed increases of miR-122 in the serum 2 h before an increase of AST/ALT [23]. Additionally, miR-122 and liver enriched miR-192 were increased in the serum of patients who had acetaminophen induced hepatotoxicity demonstrating the potential for these miRNAs to be used as both preclinical and clinical biomarkers for liver injury [24]. In a further study miR-122, HMGB1, Cytokeratin 18 and GLDH were able to predict patients who would develop liver failure as a result of acetaminophen overdose even when ALT remained in the normal range [25].

While several miRNA tissue profiling experiments have been reported in mouse, rat and human, these rodent experiments do not encompass the range of tissues relevant to toxicology studies for drug candidates. In addition, published studies relied upon microarray and/or qPCR based technologies which could suffer from a lack of specificity and sensitivity. Microarrays also do not allow for the identification of novel miRNAs due to their dependence on known sequences and may not account for tissue expression of isomiRs, which are miRNAs that have variations from the reference miRNA sequence [26]. QPCR also lacks specificity in cases where 2 miRNAs have a closely aligned sequence. As an example, miR-1 expressed in skeletal muscle and heart and miR-206 expressed specifically in skeletal muscle differ by only 3 bases. Several of the current qPCR based assays for miR-1 also detect synthetic miR-206 (Author observations) indicating that caution must be taken when using this approach.

Next generation sequencing (NGS) is a preferred method for miRNA profiling because it makes direct, unbiased and quantitative measures of known and unknown miRNAs. In order to realize the true potential of miRNAs as biomarkers of organ damage it is critical to comprehensively understand the tissue expression pattern of miRs and isomiRs, and this can be best achieved using NGS. Therefore we have constructed a rat miRNA body atlas using Illumina miRNA sequencing from 5 male and 5 female Sprague Dawley rats and 21 and 23 organs of toxicologic interest from each sex, respectively . In parallel to this effort, several of the authors have constructed a similar canine body atlas from 16 tissues from 5 male Marshall beagle dogs. Publication of both miRNA body atlases will provide an invaluable resource for the characterization of potential biomarkers of organ injury in 2 most commonly used species for preclinical safety assessment.

Here we present the findings of the rat miRNA body atlas in which we have identified 1,162 previously unreported miRNAs in the rat and detailed numerous tissue specific and enriched miRNAs. We have discovered several novel tissue specific miRNAs in each species, some of which are conserved across both rat and dog, and some which are species specific . Comparison of the rat and dog atlases yielded candidate liver and pancreas specific biomarkers. The potential pancreas biomarkers were tested in rat and dog toxicology studies using the pancreatic toxicant caerulein, while the potential liver biomarkers were tested in toxicology studies conducted by Takeda. In these proof-of-concept studies, miRNAs performed as well as or better than traditional markers of pancreatic injury, demonstrated a much larger dynamic range and increased tissue specificity over traditional biomarkers 1.

## Methods

### Necropsy

Five male and five female Sprague Dawley rats 12–13 weeks in age were obtained from Charles Rivers Laboratories and euthanized by CO_2_ asphyxiation. The rats were necropsied and tissues were placed into RNAlater® within 5 min of asphyxiation. Organs (liver, kidney, kidney medulla, kidney cortex, heart, pancreas, adrenal, glandular stomach, non-glandular stomach, duodenum, jejunum, ileum, cerebrum, cerebellum, hippocampus, brainstem, dorsal root ganglion, soleus, biceps femoris, testis, ovaries, uterus and whole blood) were processed in batches according to approximate tissue size as follows and placed into RNAlater® at 4 °C for 24 h and were transferred to-20C the following day. In batch #1, greater than 150 mg of liver, stomach, ileum, jejunum and duodenum were placed into RNAlater® for subsequent total RNA isolation. The following portions of the intestine corresponding to the segment that is routinely collected for histology were collected: the stomach with approximately 5 cm of the duodenum attached, a 10 cm section from the midpoint of the jejunum and the distal 5 cm of ileum. A syringe containing RNAlater® was used to flush the contents from the lumen of the collected segments prior to placing them into the RNAlater® collection tube. In batch #2, ~150 mg of kidney, pancreas, brain, testis, biceps and soleus were placed into RNAlater® for subsequent total RNA isolation. In batch #3, 30-50 mg of adrenal, heart, ovary and uterus were placed into RNAlater® for subsequent total RNA isolation.

### RNA Extraction

**Fig. 1:**
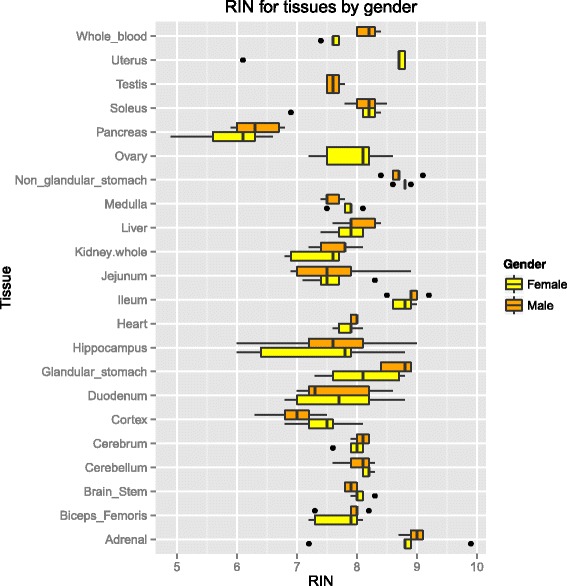
The tissues used for construction of the rat miRNA body atlas and boxplots of the RIN values associated with the tissues from 5 male and 5 female Sprague Dawley rats are illustrated above. RNAs were extracted from rat tissues and were tested for RNA quality using the Agilent bioanalyzer. Most RNAs were of very high quality as judged by RIN values. Pancreas RNAs had the lowest RIN values and also had the fewest sequences that mapped to mature miRNAs

Tissues were removed from RNAlater®, frozen in liquid nitrogen and pulverized according to the Covaris tissue pulverization protocol [27]. Tissues were placed in an appropriate volume of Qiazol and homogenized using lysing D matrix tubes (MP Biomedicals, Cat# 6913–100). Qiazol volume was scaled up according to the mass of tissue to be homogenized. Total RNA was next isolated and eluted into 80ul of 95 °C molecular biology grade water. RNA quality was assessed using the Agilent 2100 Bioanalyzer using the RNA pico kit. All RNAs except for pancreas, 3/10 hippocampus samples and 1 uterus sample had RIN (RNA Integrity Number) values of 7 or higher with an average RIN value of 7.88. For samples that displayed RIN values lower than 7 the RNA extraction protocol was repeated using additional Qiazol/Tissue homogenate. Most samples achieved RIN values greater than 7 as shown in Fig. 1. Whole blood was placed into RNAlater® for blood and all RNAs were isolated using the miRNEasy kit from Qiagen following the manufacturer’s instructions. Dorsal Root Ganglion RNAs were provided by Takeda Pharmaceuticals. RNAs were quantitated using a nanodrop and, if necessary, were concentrated by speed vac to obtain an appropriate concentration for library preparation prior to sequencing.

### miRNA seq library construction

Library construction was performed according to Illumina’s TruSeq small RNA sample prep protocol (Illumina, San Diego CA). Library quality was assessed using the Agilent Bioanalyzer DNA chip and quantified using Ribo Green. Libraries were subjected to MiSeq analysis to ensure library quality. Body atlas libraries were assigned to gels and lanes on the flow cells in a manner designed to mitigate batch effect. Subsequent HiSeq analysis was performed and 50 bp single end reads were generated on the Illumina HiSeq 2000 platform resulting in 4–5 million reads per sample.

### Data analysis by institution

#### Read mapping and quantification

Data was analyzed by Eli Lilly, Maastricht University and NIEHS using separate analysis methods. The output generated from each institution was then collated to create a Venn diagram of the tissue enriched miRNAs identified by each analysis method. Read mapping and quantification for the rat and dog miRNA body atlases was performed in the following manner by Eli Lilly: FastQ files were processed to remove adaptor sequences and discard reads < 17 bp in length with cutadapt v. 1.4.1 [28] with options –a TGGAATTCTCGGGTGCCAAGG–quality–base 33–q 20–match-read-wildcards –m 17. All trimmed reads that contained an ‘N’ were discarded. Identical sequences from the same sample were combined into a single sequence in the form expected by miRDeep2 [29]. That is, with ids of the form “NNN_#_x#”, where NNN is a unique alphabetic code identifying the sample, the first ‘#’ is a unique integer, and ‘x#’ records the number of times this sequence occurred in the input for the sample. Quantifier.pl from the miRDeep2 package v. 2.0.0 was used to generate miR alignment files (.mrd files) against known miRs from miRbase 20 with options–P–p < org > _hairpin.fa –m < org > _mature.fa–r trimmed_reads.fa–d. This was done separately for rat, mouse, human, and *C. elegans* known miRs. The.mrd files were then parsed with a custom Perl script as follows. Each isomiR sequence in an alignment was associated with the corresponding mature miR identifier. If it aligned to a miRNA precursor, but not with an expected mature miR sequence, it was identified as < miR > −pre to indicate this. Usually these correspond with reverse strand miRs that have not yet been annotated as alternate mature forms. Sometimes these appear to be microRNA offset RNAs [30], and sometimes they appear to represent incompletely processed sequence. If a given sequence was identified as aligning with more than one precursor, it was associated with all potential names as a composite name. That is, a sequence that aligned to both let-7a-5p and let-7f-5p was assigned to the composite mature miR let-7a-5p;let-7f-5p to indicate for subsequent analyses that this identification was ambiguous. All sequences that had not yet been identified against known rat miRs were then looked for in the analysis with respect to known mouse miRs, and so on against human and finally *C. elegans* miRs. This process allows for identification of conserved rodent miRs that have not yet been annotated in rat, and against conserved mammalian miRs that have not yet been annotated in rat and mouse. In parallel to the above identification of known miRs, we used miRDeep2’s novel miR identification process. Additional details are described in Additional file 1: supplementary methods.

#### Tissue specific and enriched miRNA identification

Data analysis of the rat and dog sequencing data was performed by Eli Lilly as follows. Tissue specific miRNAs are defined as miRNAs that are expressed at a raw count of greater than 1 in 8 out of 10 rats in 1 organ/ tissue only. For example, the brain may be the only organ with miR-X expression, but the hypothalamus may be the only tissue that has miR-X expressed. Therefore, miR-X would be specific to the hypothalamus tissue. Tissue enriched miRNAs are defined as miRNAs that are expressed at a raw count of greater than 1 in 8 out of 10 rats in a few organs/tissues, typically 3–5 tissues. The definition of tissue enriched was purposely vague because a miRNA may be enriched in several organs, but could still be a useful biomarker if toxicity is only present in 1 of the organs the miRNA is enriched in. Sex specific miRNAs are identified as miRNAs that are expressed in 1 tissue/organ at a raw count of greater than 1 in 4/5 males or females and in 0/5 rats in the opposite sex. Raw and normalized data were analyzed for tissue specific and tissue enriched miRNAs. Animal level data is obtained after preprocessing of the raw sequencing data. For each microRNA, there are measurements from 10 animals (5 males and 5 females) and potentially 23 different tissues. These 23 tissues were from 14 organs. In order to find tissue specific miRNAs, tissue level counts were obtained for each microRNA by summing over all the animals. The tissue level counts were aggregated to organ level by selecting the maximum of the tissue counts to represent the organ count. For example, brain (organ) has 4 different tissue types (Hippocampus, Brainstem, Cerebellum, and Cerebrum), the maximum of these 4 tissue level counts will be used to represent the expression levels of brain. After these data processing steps, a count data matrix is generated, where each row corresponds to a miR and each column corresponds to an organ type.

Non-negative Matrix Factorization (NMF) [31] is a commonly used unsupervised learning algorithm to decompose a nonnegative matrix X into 2 non-negative matrices W and H. In our case, X is the observed count matrix of dimension Nx14, where N is the number of miRs. Each column of W defines a miR group and each column of H represents the expression pattern of the miR group corresponding to the organ type. Based on this decomposition, we can find a representative set of miRs that corresponds to the specific high expression pattern for the organ.

To identify tissue specific miRs, it has to be organ specific (which means that it has to be highly expressed in 1 organ, and lowly in the rest of the organs). Thus we start from each organ specific miR, and examine if it is also highly expressed in a particular type of tissue or a few tissues, which belong to the current organ. For example, a miRNA may be specific to brain (organ), but it may only be specific to the hippocampus (tissue). To determine the expression level of each miR, we fitted a two-component mixture of Poisson distribution to the animal level tissue counts data. The component with larger intensity of the Poisson distribution is naturally used to model high counts and the component with lower intensity is used to model background noise. The larger component of the Poisson mixture model naturally corresponds to high expression level, whereas the smaller component of the Poisson mixture model naturally corresponds to the low expression level. For an organ specific microRNA to be tissue specific, it has to be expressed at a high level in 1 tissue of this organ. After the mixture model is fitted and the intensities are estimated, we can determine which tissues the microRNA is expressed in according to their estimated intensities and then declare tissue specific microRNAs afterwards. Additionally, the raw sequencing data was manually analyzed in each tissue for each miRNA and statistical analysis of the manually identified tissue specific and enriched miRNAs was then conducted to verify or dispute the tissue specificity or enrichment.

#### Read mapping and quantitation by Maastricht University

Read mapping and quantification was performed in the following manner by Maastricht University: mirDeep2 [32] was used to predict putative novel miRNAs. The raw fastq files from the 215 rat samples were mapped to the Rat genome (version 5.0.73 from Ensembl). MiRDeep2 output was then parsed to keep only the 496 predicted miRNA precursors with a score of 1 or above. Raw reads were trimmed off the 3′ adapter sequence using the java algorithm “Adaptor Remover” from the freely available UEA sRNA Workbench (http://srna-workbench.cmp.uea.ac.uk/). The parameters were set to look for a perfect match with the first 8 bases of the 3′ adapter to be considered a hit. The trimmed sequences with a size below 16 or above 35 bp were discarded. During this trimming step, every identical sequence was pulled and each iteration was summed. The trimmed reads were then mapped to the list of rat precursor miRNA obtained either from miRBase, or generated *de novo* from the miRDeep2 prediction using a fast short read mapping software PatMaN [33], configured to allow no mismatches or gap. PatMan output was then parsed to 1) assign a unique name to each unique sequence consisting of the pre-miRNA name, the pre-miRNA arms (5p or 3p) and the mapping coordinates (for instance, miR-127_3p_57_78). 2) divide the total read count of each unique sequence by the number of assigned loci in the complete mapping. 3) create a mature output table by pulling the different isomiR count for each miRNA species (i.e. 5p and 3p). The parsed PatMaN output were normalized with the trimmed mean of M-values method (TMM) and filtered to remove all miRNAs where the normalized read counts were fewer than 10 reads in all the 215 samples. Total read counts were then computed for each individual miRNA.

#### Tissue enriched miRNA identification by Maastricht University

A miRNA was called tissue specific or tissue enriched when the percentage of reads for a single tissue (or tissue group) was respectively higher than 90 or 50 % of the total read count.

#### Read mapping and quantitation by NIEHS

In order to identify isomiRs the raw count number of each given isomiR was first converted to the percentage of expression compared to the mature count observed for its corresponding miRNA. By comparing this percentage, we were able to report the miRNA for which the most expressed isomiRs differs between tissues.

Analysis of the data performed at NIEHS was executed in the following manner: Raw fastq files with the miRNA-Seq reads were checked for quality using a **TQE** standard based on **t**rimming the adapters, **q**uality filtering at Q ≤ 20 and the **e**limination of short reads < 14 bases long. In some cases when minor adapter sequences (indices) were observed after initial process, additional rounds of processing were applied until all the criteria were met. In the end, reads longer than 25 base pairs were also eliminated, resulting in quality filtered reads between 14–24 bp. Reads passing the **TQE** filtering were then aligned to the rat miRBase database v.19 using BWA version 0.6.1-r104q with “-n 2” and default parameters otherwise. Only perfect matches were further used in the quantification, which was performed by summarizing read count level measurements at each mature miRBase miRs. Datapoints with zeros were imputed with 1.0. Total counts for each miR were normalized by transcripts per million reads (TPM). Prior to statistical analysis, a transformation from floats to integers was performed by ceiling the data.

#### Tissue enriched miRNA identification by NIEHS

To detect the tissue-enriched microRNAs, we adopted the one-vs-rest strategy when applying statistic models. “Differentially” expressed miRs (DEMiRs) in 1 tissue against the combination of all other tissues were identified using a quasi-Poisson approach (Quasi-Seq) with shrunken dispersion estimates [34]. During the modeling process, the offset was computed on the 3rd quartile. Significant DEMiRs were detected at a nominal *p*-value < 0.01.

### Rat and dog toxicity studies

Caerulein studies: Studies were run at Covance Laboratories in Greenfield, IN and histopathology was performed by a board certified pathologist. Due to the short half-life of caerulein [35], 6 male Sprague Dawley rats per group (obtained from Charles Rivers laboratories 11–12 weeks at study start) were treated with 3 intraperitoneal doses of vehicle or caerulein (Sigma Aldrich), 1 h apart, at 0, 15 and 50 μg/kg. Blood was collected at 1, 4, 8, 24 and 48 h after dosing and examined for clinical chemistry, hematology and microRNA changes with terminal serum taken at 8, 24 and 48 h. Urine was collected by cystocentesis at 0, 4, 8, 24 and 48 h and examined for total protein and specific gravity. Toxicokinetic analysis was performed at 0.5, 1, 4, 8, 24 and 48 h post dose. Histopathology of adrenal glands, bone (sternum), brain stem, cerebellum, cerebrum, duodenum, heart, ileum, jejunum, kidneys, liver, lung, muscle (plantaris), pancreas, spleen, stomach and thymus was examined at 8, 24 and 48 h post dose. For canine studies, 2 beagle dogs per group, 1 male and 1 female obtained from Covance Research Products, were given 3 doses 1 h apart of caerulein at 3, 15 or 45 μg/kg. Blood was collected at pre-dose, 1, 4, 8, 24, 48 and 72 h after the third dose and examined for clinical chemistry, hematology and miRNA changes. Urine was collected by cystocentesis and examined for changes in specific gravity, BUN, creatinine and other urinary parameters at necropsy at 24 and 72 h. Histopathology on organs including adrenal glands, bone (sternum), brain stem, cerebellum, cerebrum, colon, epididymis, eyes, heart, ileum, jejunum, kidneys, liver, lung, muscle (quadriceps femoris), sciatic nerve, ovaries, pancreas, prostate, spleen, stomach, testis, thymus, thyroid, uterus and vagina was performed at 24 and 72 h post dose. Animals were euthanized by barbiturate overdose or anesthesia and exsanguination at necropsy.

### qPCR of serum samples from caerulein studies

For each sample, 100 μl of serum from all studies was subjected to total RNA isolation, including miRNAs, using the miRNeasy ^TM^ kit from Qiagen (Valencia CA). Upon the addition of Qiazol 5 μl of 5 μM synthetic cel-miR-55-3p (IDT) was spiked into each sample in the rat caerulein and dog caerulein studies. RNAs were eluted in 80ul of 95 °C H2O and a water extraction control with spike-in was also included. Reverse transcription was executed with the miRCURY LNA microRNA Universal cDNA Synthesis Kit (Copenhagen, Denmark). 10 μl of the extracted RNAs were used in 20 μl RT reactions. A no template control (NTC) and two -RT controls were also included. Real-Time PCR was conducted using Exiqon (Additional file 2) primers and ExiLENT SYBR Green Master Mix (Copenhagen, Denmark). Samples were diluted 1:40 prior to qPCR and 5 μl of the diluted samples were used in 10 μl reactions. Data was collected on a 7900HT Real-Time PCR instrument (Life Technologies, Grand Island, NY).

### Statistical analysis of rat and dog caerulein qPCR data

For each study, the fold change data were log2 transformed and fitted to a two-way ANOVA model with repeated measures. Because each animal has multiple measurements, we allow these measurements to be auto-correlated. Once we obtained the parameter estimates, for the dog study, all treatment means and 95 % confidence intervals at each time point are compared against the pre-dose group, which have FC fixed at 1. For the rat study, low dose and high dose groups are compared to untreated group at each time point.

### qPCR validation of tissue specific and enriched miRNA

Tissue enriched and tissue specific miRNAs were chosen for qPCR validation. The RNAs from 3 rats and 21 tissues were normalized by mass (1 ng of RNA per tissue) and subjected to cDNA synthesis using Exiqon universal cDNA synthesis kits (Cat# 203301) and ExiLENT SYBR green mastermix (Cat# 203421). Data was collected on a 7900HT or ViiA7 Real-Time PCR instrument (Life Technologies, Grand Island, NY). Assays were designed to the most highly expressed miRNA or isomiR sequence present that mapped to the mature miRNA. For example, many isomiRs mapped to miR-XYZ. For qPCR analysis we designed primers to the most highly expressed miRNA sequence that mapped to miR-XYZ as determined by deep sequencing. Ct values of each miRNA were compared to one another to determine tissue specificity or enrichment. A Ct value was considered acceptable if the Ct in a particular tissue was 10 Cts lower than the signal in the no RT or no template (NTC) controls unless there was no signal in the no RT or NTC. If an assay did not display a signal that was not 10 Cts away from the no RT or NTC it was not included in the analysis. Additionally, if the melt curves for an assay displayed more than one peak the assay was not included in the analysis.

### qPCR analysis of pancreas enriched miRNAs

Total RNA from all male and female rats used in the study were normalized by mass using 250 ng of RNA per sample and subjected to cDNA synthesis using Exiqon universal cDNA synthesis kits (Cat# 203301) and ExiLENT SYBR green mastermix (Cat# 203421). Data was collected on a 7900HT or ViiA7 Real-Time PCR instrument (Life Technologies, Grand Island, NY).

## Results

**Fig. 2:**
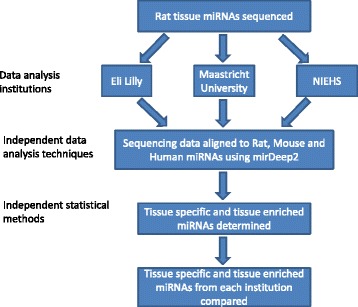
Workflow diagram. The small RNA content of rat organs was interrogated by deep sequencing and the data was given to 3 institutions for independent data analysis. Reads were mapped to rat, mouse and human miRNAs from miRBase v19 and v20 using miRDeep2 and tissue specific and tissue enriched miRNAs were identified

Our first objective was to identify miRNAs expressed in rat tissues. Members of the HESI consortium were queried on which rat organs are most commonly injured during toxicology studies and therefore, would represent the most important organs to investigate for potential biomarkers of injury. This resulted in the list of organs detailed in Fig. 1. Dorsal root ganglion was added as an organ of investigative interest. The brain lacks serum and cerebrospinal fluid (CSF) based biomarkers of injury, therefore the miRNA content of the cerebrum, cerebellum, hippocampus and brainstem were interrogated. Additionally, whole blood was included in the study in order to determine what the contribution of blood miRNAs may be to highly vascularized tissues. Total RNA was isolated from the tissues of male and female SD rats and the quality was assessed using RIN values (Fig. 1) to be sure that small RNAs would be sequenced as opposed to degraded RNAs. The rat microRNA sequencing data, as analyzed by Eli Lilly, generated 1,927 sequences that mapped to previously-identified mature microRNAs in rat, mouse and human. This resulted in an increase of 1,162 miRNAs in the rat as compared to the 765 mature rat miRNAs in miRBase v21. While Eli Lilly’s data analysis serves as the main source of data for discussion in this article we also collaborated with HESI members to analyze the data in an attempt to maximize our ability to discover and confirm the tissue enrichment profile of miRNAs (Fig. 2).

**Fig. 3:**
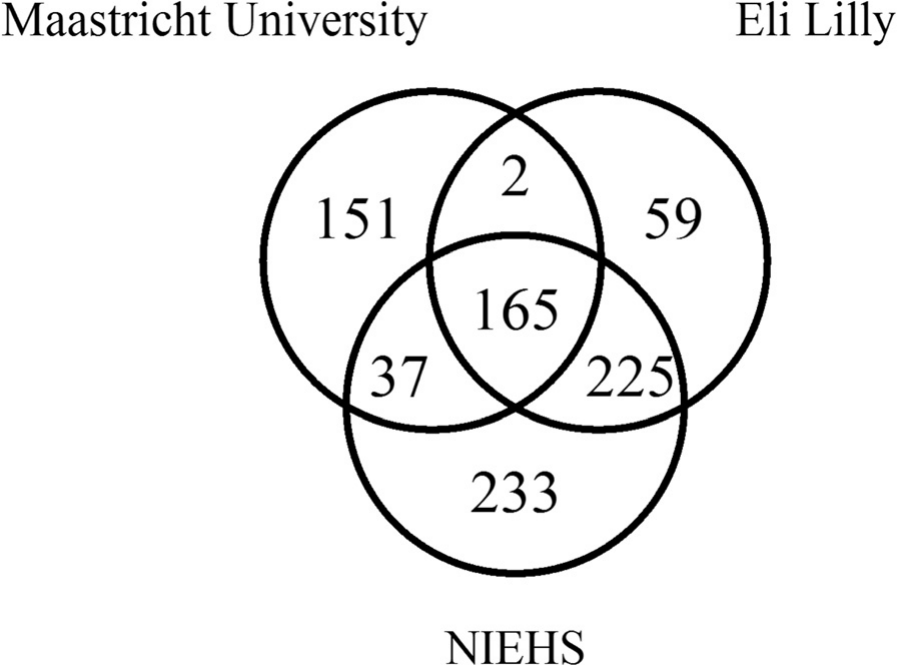
Venn diagram of data analysis methods. Analysis of the miRNA sequencing data was independently performed by Eli Lilly, Maastricht University and the NIEHS using different data analysis methods. Eli Lilly identified 481 tissue enriched miRNAs, Maastricht University identified 355 tissue enriched miRNAs and the NIEHS identified 660 tissue enriched miRNAs. The number of miRNAs in common between Maastricht University and Eli Lilly was 167, the number of miRNAs in common between Maastricht University and the NIEHS was 202 and the number of miRNAs in common between Eli Lilly and the NIEHS was 401. The number of miRNAs in common between all 3 analysis was 165

Our second objective was to discover novel and confirm previously identified tissue specific and/or tissue enriched miRNAs and/or isomiRs that could be used as serum based biomarkers of organ injury in the rat. A variety of data analysis software packages and statistical methods exist to identify and predict miRNAs and perform differential expression on miRNA-seq data [36, 37]. Since these methods have different strengths and weaknesses and the most suitable data analysis method is dependent on the output required, different methods were employed by scientists at Eli Lilly, NIEHS and Maastricht University to analyze the sequencing data in order to maximize discovery of tissue enriched miRNAs. The tissue enriched miRNAs identified by each institution were included in a Venn Diagram in order to determine which tissue enriched miRNAs were found in common between each data analysis method (Fig. 3). Tissue specific miRNAs identified by Lilly are reported in Table 1 and were not included in the Venn diagram. Maastricht University identified 355 tissue enriched miRNAs, Eli Lilly identified 481 tissue enriched miRNAs and NIEHS identified 660 tissue enriched miRNAs. Maastricht University and Eli Lilly had 167 miRNAs in common, Maastricht University and NIEHS had 202 miRNAs in common, Eli Lilly and NIEHS had 401 miRNAs in common and all 3 institutions had 165 tissue enriched miRNAs in common (Additional file 3: Table S1). Differences in the results from each institution are due to the processing steps prior to alignment, the reference sequences the sequencing data were aligned to and the methods of identifying tissue specific and/or enriched miRNAs. Despite which approach was taken each analysis identified many of the same tissue enriched miRNAs and the data has been made publically available for researchers in industry, academia or government to explore and potentially discover additional tissue specific and/or enriched miRNAs which may serve as novel biomarkers for safety, pharmacodynamic or other effects.

**Table 1:** Rat tissue specific miRNAs

Tissue	miRNA
Adrenal	miR-126b, miR-494-5p, mir-134-pre, miR-3544, miR-299a-5p;miR-299b-5p
Brainstem	miR-296-3p, miR-7047-3p, miR-186-3p
Cerebellum	mir-1928-pre, mir-484-pre, miR-130a-3p;miR-130b-3p, miR-6125, miR-154-3p
Cerebrum	miR-218-5p, miR-466i-5p;mir-466 k-pre;mir-466q-pre, miR-410-5p, miR-344 g, mir-504-pre, miR-344b-1-3p;miR-344b-2-3p, miR-551b-5p, miR-103-2-5p
Heart	miR-208a-3p, miR-208a-5p, miR-126-3p, miR-6314, miR-509-3p
Ileum	miR-2137, miR-3195, miR-320-5p, miR-4448
Liver	miR-101b-5p, miR-122-3p, miR-1195, miR-466c-3p, miR-293-5p, miR-466b-2-3p;miR-466c-3p;mir-466d-pre, miR-692, miR-466b-2-3p, mir-328-pre, miR-466f-3p, mir-5131-pre
Medulla	miR-200b-3p;miR-429
Muscle Biceps	miR-675-5p
Muscle Soleus	miR-206-5p, miR-1273 g-3p;mir-1273a-pre
Pancreas	miR-320c, miR-148b-3p;miR-152-3p
Whole Blood	mir-466q-pre, miR-7687-5p, miR-3656;mir-3195-pre, miR-3473b;mir-3473d-pre, miR-6937-5p

Tissue specific miRNAs were defined as miRNAs there are expressed at a raw count of >1 in 8/10 rats in 1 tissue only. Sequencing data was analyzed by non-negative matrix factorization which identified tissue specific miRNAs. Organs not listed in the table did not have tissue specific miRNAs

**Fig. 4:**
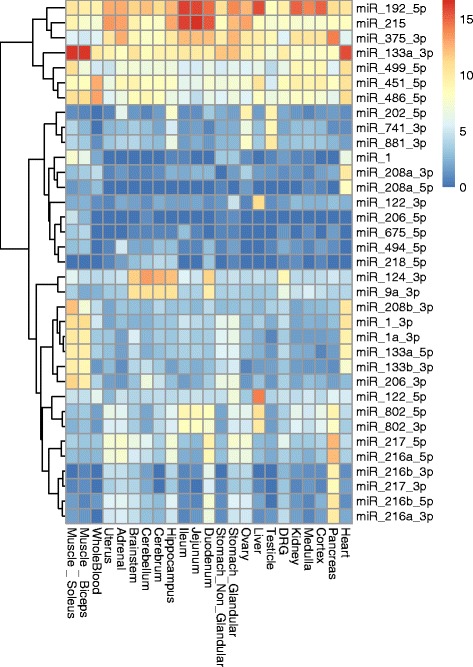
Heat map of tissue specific and tissue enriched miRNAs. Examples of tissue specific and enriched miRNAs identified by this and previously published studies are displayed with tissues on the x-axis and miRNAs listed on the y-axis. MiRNA expression from least to highest expression is indicated as a shift from blue to red with blue representing the least expression and dark red indicating maximal expression

**Fig. 5:**
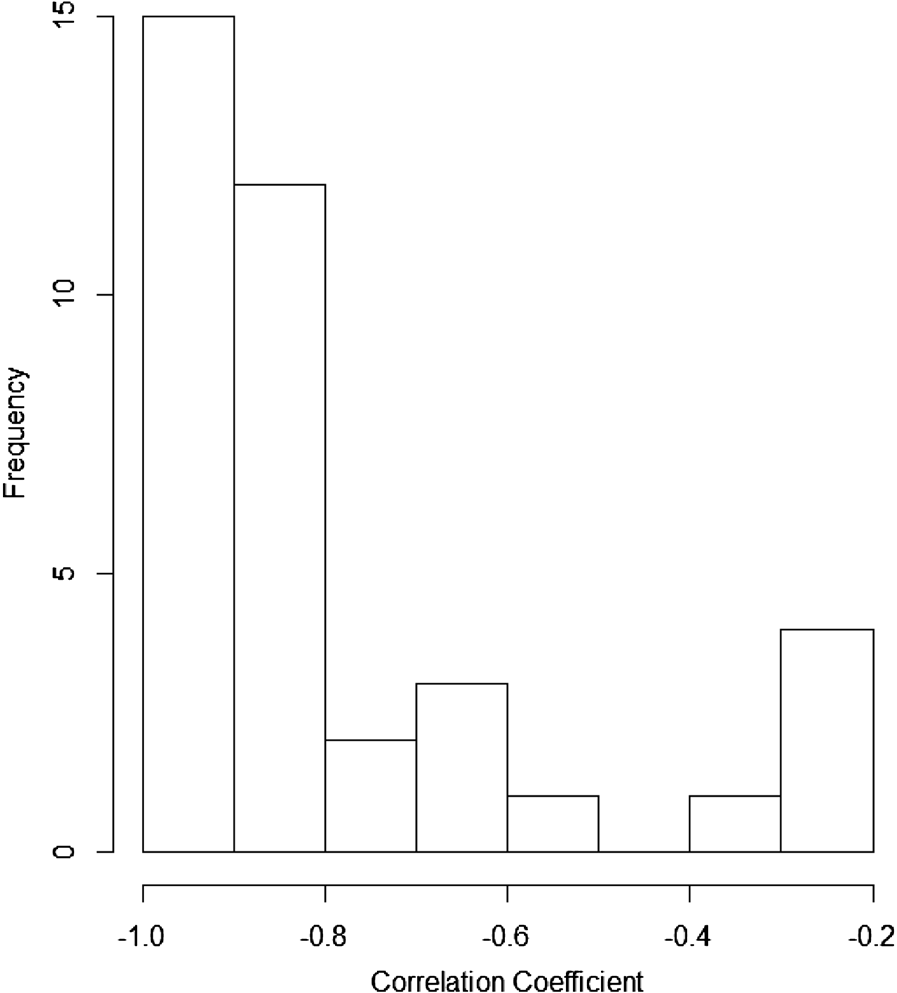
Correlation of qPCR analysis of tissue specific and/or enriched miRNAs. Tissue specific and/or enriched miRNAs were chosen according to tissues that lack or require additional biomarkers of organ status and were measured in 21 tissues from 3 female rats by qPCR. The qPCR data was compared to the sequencing raw counts for each miRNA. A perfect correlation is equal to − 1 because the Ct value decreases as sequencing abundance increases. Twenty seven of the thirty nine miRNAs tested correlated very well to the sequencing data with a correlation of − 0.8 to − 1.0, 8 miRNAs displayed a correlation of − 0.79 to − 0.57 and 4 miRNAs displayed poorer correlations from − 0.33 to − 0.22. MiRs-196c-5p and 206-5p were not amplified by qPCR and, therefore, did not display tissue specificity or enrichment by qPCR

Tissue specific and enriched miRNAs identified by Lilly are shown in (Additional file 4: Table S2) and (Additional file 5: Figure S1) and the tissue enriched miRNAs identified by NIEHS and Maastricht University are shown in (Additional file 6: Table S3 and Additional file 7: Table S4 respectively). The tissue specificity/enrichment of many other tissue enriched miRNAs including muscle and heart enriched miRs-133a-3p, 499-5p, miR-1a-3p as well as pancreas enriched miRs-216a-5p, 217-5p and miR-375-3p were confirmed [38–40] (Fig. 4). The brain, testis and liver have the most tissue specific and enriched miRNAs and interestingly, tissue enriched miRNAs in the brain are often expressed in the intestine. The kidney has the fewest tissue specific and enriched miRNAs of any organ examined, while skeletal muscle and heart share several of the same tissue enriched miRNAs. While previously identified tissue specific and enriched miRNAs were verified, previously unidentified tissue specific/enriched miRNAs were discovered including, but not limited to, pancreas enriched miRs-148a-3p, 141-3p and 200c-3p (Additional file 4: Table S2). In order to verify that miRNAs were, in fact, tissue specific or enriched we conducted qPCR verification of some of the tissue specific and/or enriched miRNAs (Additional file 8: Table S5). As shown in Fig. 5, 27 of the 39 miRNAs tested correlated very well to the sequencing data with a correlation of − 0.8 to − 1.0, 8 miRNAs displayed a correlation of − 0.79 to − 0.57 and 4 miRNAs displayed poorer correlations from − 0.33 to − 0.22 (Fig. 5 and Additional file 9: Table S6). MiRs-196c-5p and 206-5p were not amplified by qPCR and, therefore, did not display tissue specificity or enrichment by qPCR.

While each institution was able to identify tissue specific and enriched miRNAs it is also imperative to understand whether the isomiRs of the tissue specific and enriched miRNAs are expressed in the same tissues as the parent miRNA as isomiR expression in other tissues could lead to inaccurate interpretation of serum miRNA profiles with respect to toxicity. Eli Lilly investigated the isomiR expression of pancreas tissue specific and enriched miRNAs and the analysis revealed that isomiRs generally mirror their parent miRNA expression (Additional file 10: Figure S2), but some isomiRs are more tissue specific than others as shown for miR-215 in the intestines (Additional file 11: Figure S3) and miR-217-5p in the pancreas (Additional file 10: Figure S2). For example, the mature miR-217-5p sequence is 5′TACTGCATCAGGAACTGACTGG-3′ and the isomiR-217-5p sequence 5′-TACTGCATCAGGAACTGACTGGAC-3′ are both highly enriched in the pancreas, but the miR-217-5p isomiR lacks expression in the stomach, intestine and ovary while other isomiRs of miR-217-5p are expressed in the stomach, intestine and ovary (see the last miR-217-5p isomiR in Additional file 10: Figure S2). IsomiRs in other tissues such as isomiRs of miR-215 in the intestine (Additional file 11: Figure S3), miR-192-5p in the liver (Additional file 12: Figure S4) and miR-3473f-pre in the testis (Additional file 13: Figure S5 and Additional file 14: Figure S6) display similar isomiR expression. It appears that interpretation of serum miRNA toxicity biomarker data should not be compromised by expression of isomiRs from tissues lacking parent miRNA expression.

### Rat and dog Pancreas toxicity studies

**Fig. 6:**
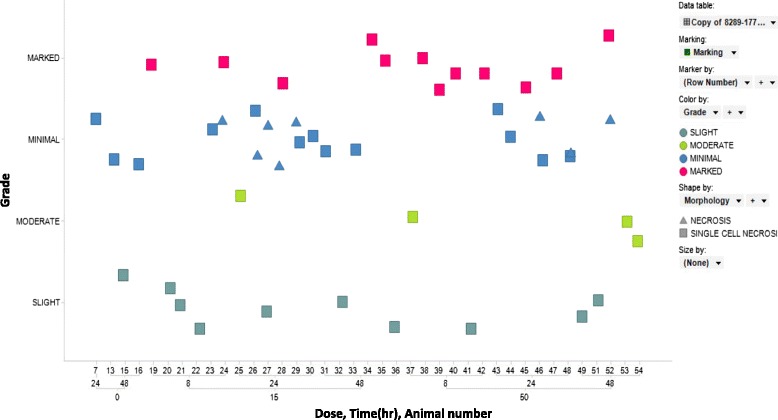
Histopathologic assessment of the pancreases of rats treated with 15 and 50ug/kg of caerulein. Histopathologic assessment of organs was performed at 8, 24 and 48 h with dose, time and animal number indicated on the x-axis and the grade of injury indicated on the y-axis (grey-blue indicates slight injury, green indicates moderate injury, blue indicates minimal injury and pink indicates marked injury). Rat pancreases displayed minimal and slight pancreatic necrosis in 4 vehicle treated rats at 24 and 48 h while dose responsive increases in the severity of necrosis was noted in the 15 and 50ug/kg treatred rats at 8, 24 and 48 h

**Fig. 7:**
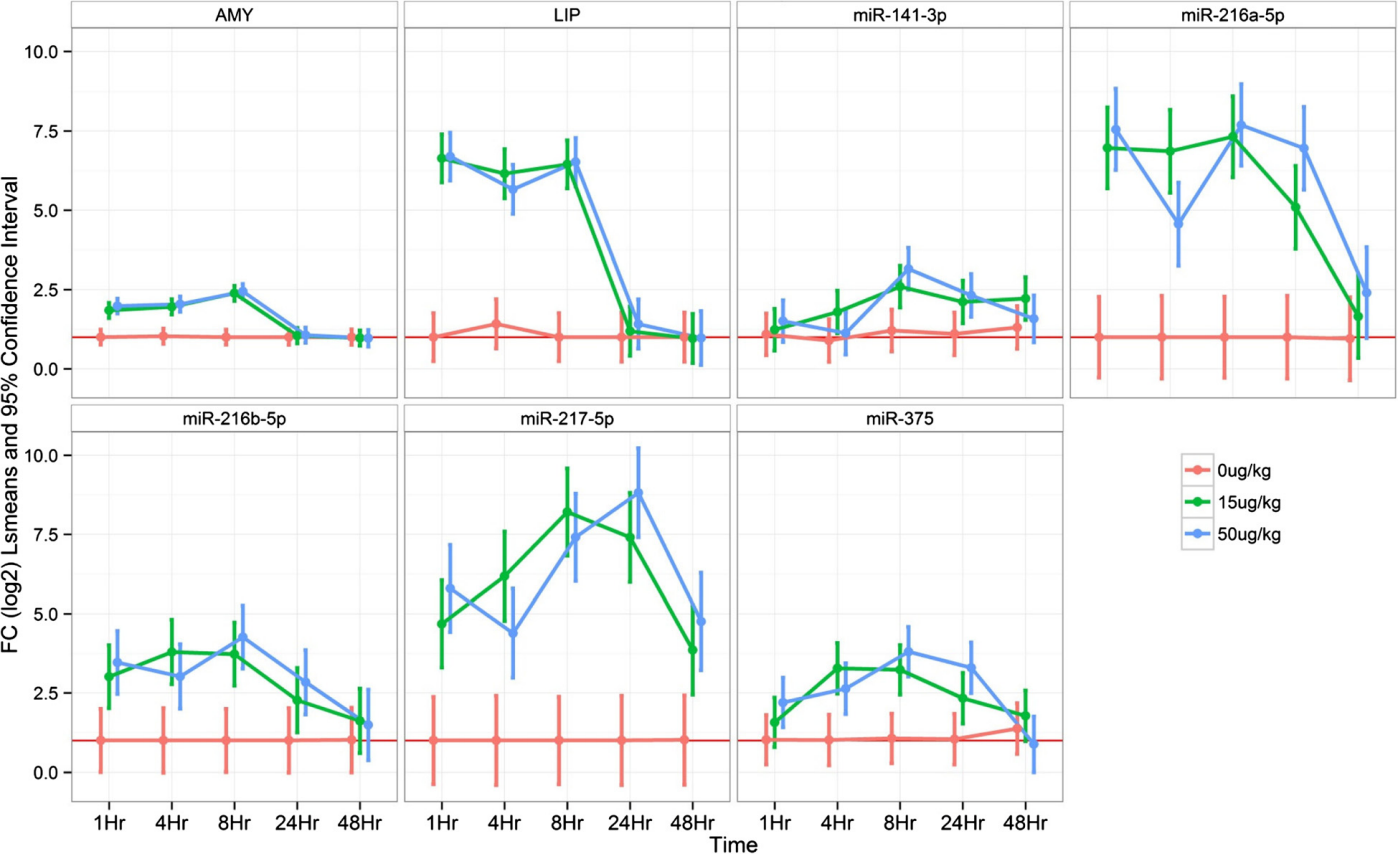
Clinical chemistry and serum miRNA analysis of caerulein treated rats. Rats treated with 0, 15 and 50ug/kg of caerulein were examined for clinical chemistry and miRNA changes (doses are indicated by red, green and blue respectively). Time is indicated on the x-axis and the log 2 of the fold change, the mean as well as 95 % confidence interval are indicated on the y-axis. miRNAs were incereased in the serum of rats with 15 and 50 ug/kg of caerulein coincident with increases in amylase and lipase with maximal increases occurring at 8–24 h for miRs-216a-5p, 217-5p and 375-3p. Values are normalized to vehicle and to the cel-miR-55-3p spike in

Conserved pancreas tissue enriched miRNAs were examined in the rat and dog models of caerulein pancreatic toxicity in order to begin to characterize miRNAs as species translatable serum based biomarkers of pancreatic injury (Table 2). Rats were treated with vehicle, 15 and 50 μg/kg of caerulein and the serum was examined for clinical chemistry and miRNA changes at 1, 4, 8, 24 and 48 h with histopathologic assessment of organs at 8, 24 and 48 h. Minimal and slight pancreatic necrosis was noted in 4 vehicle treated rats at 24 and 48 h which had no clinical chemistry or miRNA correlates and were considered to be consistent with common background while minimal, slight, moderate and marked pancreatic necrosis was noted in the 15 and 50 μg/kg treated rats that was interpreted to be dose related (Fig. 6). Clinical chemistry parameters including amylase and lipase (markers of pancreas injury), miR-122-5p (liver enriched), miR-133a-3p (muscle enriched), 148a-3p (pancreas enriched), 208a-3p (heart enriched) and pancreas miRNAs conserved between rat and dog (Table 2) were examined for changes in the serum. MiR-216a-5p, amylase and lipase were increased in the serum concurrently at 1 h, remained elevated until 8 h while miR-216a-5p remained elevated until 24 h. MiR-217-5p displayed similar kinetics to miR-216a-5p except miR-217-5p generally had a larger dynamic range and remained elevated until 48 h. Both miRs-216a-5p and 217-5p displayed much larger dynamic ranges than amylase or lipase and remained elevated longer. MiR-216b-5p displayed serum increases that were kinetically similar to amylase and lipase, but had a dynamic range that was less than lipase and greater than amylase. MiR-375-3p was increased from 4–24 h in the 15 and 50 μg/kg groups and returned to approximately vehicle level by 48 h. The pancreas enriched miRNAs conserved between rat and dog (miR-101c, 141-3p, 148a-3p, 193b-3p, 200c-3p,) that were tested displayed increases in the serum similar to amylase (data not shown), whereas miRs 320-3p, 4286 and 5100 were not increased (Fig. 7 and statistical analysis Additional file 15: Table S7).

**Table 2:** Pancreas enriched miRNAs conserved between rat and dog

Rat pancreas	Sequence (5′-3′)	Dog Pancreas	Sequence (5′-3′)
miR-101c	**T**ACAGTACTGTGATAACTGA**TC**	miR-101c	ACAGTACTGTGATAACTGACC
miR-141-3p	TAACACTGTCTGGTAAAGATG	miR-141	TAACACTGTCTGGTAAAGATG
miR-193b-3p	AACTGGCCCACAAAGTCCCGCT	miR-193a-3p	AACTGGCC**T**ACAAAGTCCC**A**G**T**T
miR-200c-3p	TAATACTGCCGGGTAATGATGGA	miR-200c	TAATACTGCCGGGTAATGATGGA
miR-216a-5p	TAATCTCAGCTGGCAACTGTG	miR-216a	TAATCTCAGCTGGCAACTGTG**A**
miR-216b-5p	AAATCTCTGCAGGCAAATGTGA	miR-216b	AAATCTCTGCAGGCAAATGTGA
miR-320-3p	AAAAGCTGGGTTGAGAGGGCGA	miR-320b;miR-320c	AAAAGCTGGGTTGAGAGGG**AA**
miR-4286	ACCCCACTCCTGGTACCA	miR-4286	ACCCCACTCCTGGTACCA
miR-5100	ATCCCAGCGGTGCCTCCA	miR-5100	**GTTCGA**ATCCCAGCGGTG
miR-217-5p	TACTGCATCAGGAACTGA**CTGGA**	miR-217	**A**TACTGCATCAGGAACTGATT
miR-375	TTTGTTCGTTCGGCTCGCGTGA	miR-375	TTTGTTCGTTCGGCTCGCGTGA

Tissue enriched miRNAs conserved between rat and dog were identified and are shown above. These miRNAs were analyzed for changes in the serum in rat and dog caerulein toxicology studies. Bases underlined indicate bases that are not conserved between the rat and dog isomiRs with the highest sequencing counts

**Fig. 8:**
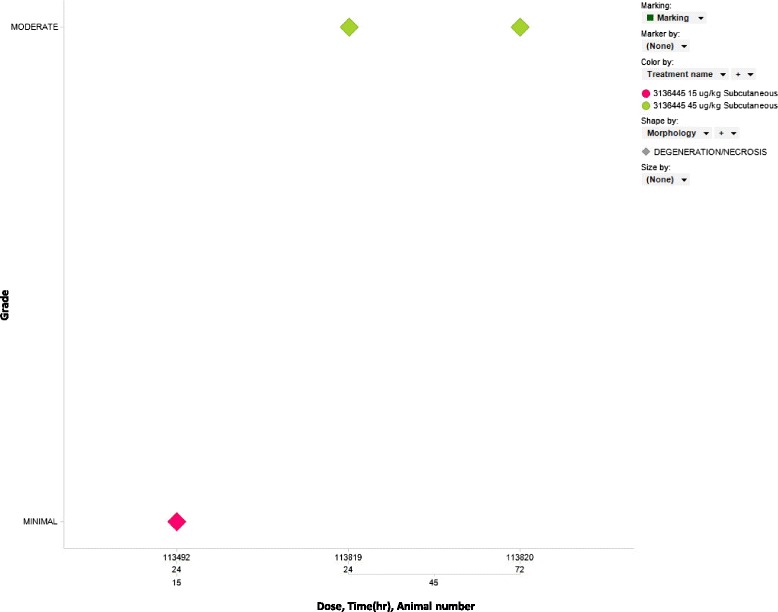
Histopathologic assessment of the pancreases of dogs treated with caerulein. Marshall beagle dogs were treated with 3, 15 and 45 ug/kg of caerulein and histopathologic assessment of organs was performed at 24 and 72 h with dose, time and animal number indicated on the x-axis and the grade of injury indicated on the y-axis (pink indicates minimal injury and green indicates moderate injury). Pancreatic degeneration/necrosis was not noted in dogs treated with 3ug/kg of caerulein. Pancreatic degeneration/necrosis was noted in 1 dog at 24 h in the 15ug/kg group and displayed dose dependent increases in severity at 24 and 72 h in the 45ug/kg group

**Fig. 9:**
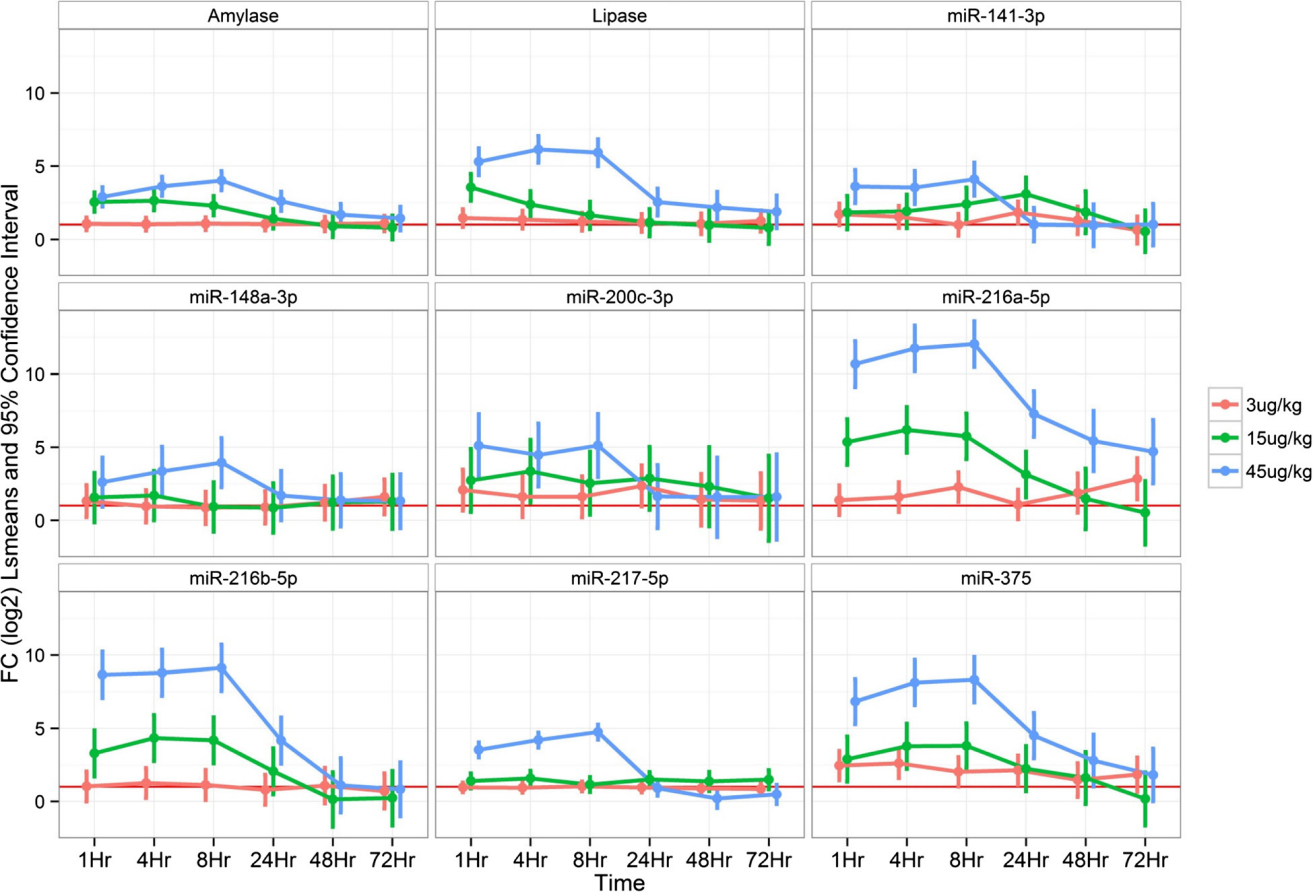
Clinical chemistry and serum miRNA analysis of caerulein treated dogs. Dogs treated with 3, 15 and 45 ug/kg of caerulein were examined for clinical chemistry and serum miRNA changes (doses are indicated by red, green and blue respectively). Time is indicated on the x-axis and the log 2 of the fold change, the mean as well as 95 % confidence interval are indicated on the y-axis.. miRNAs were increased in the serum of dogs trreated with 3ug/kg of caerulein prior to increases in amylase and lipase. miRNAs were dose dependently increased in the serum of dogs treated with 15 and 45 ug/kg of caerulein and correlated to amylase and lipase increases while miR-216a-5p remained elevated until 24 h. Values are normalized to vehicle and to the cel-miR-55-3p spike in

Marshall beagle dogs were treated with vehicle, 3, 15 and 45 μg/kg of caerulein and the serum was examined for clinical chemistry and miRNA changes at 1, 4, 8, 24, 48 and 72 h with histopathologic assessment of organs at 24 and 72 h. Pancreatic necrosis was noted in 1 dog at 24 h in the 15 μg/kg group and 24 and 72 h in the 45 μg/kg group (Fig. 8). Amylase and lipase were not increased greater than two fold in the vehicle or 3 μg/kg treated dogs at any time point. Amylase and lipase were increased in the 15 μg/kg treated dogs with the largest increases at 1 h and decreasing by 8–24 h while both were increased maximally at 4-8 h in the 45 μg/kg treated group. MiR-216b-5p was increased in 1 vehicle treated animal and miR-217-5p was increased in 2 vehicle treated animals with no histopathologic or clinical chemistry correlates (data not shown). In 3 μg/kg treated dogs miR-216b-5p and miR-141-3p were increased at some time points, but miR-216b-5p was not increased beyond the level of the vehicle treated dog and did not display consistent time dependent increases. MiR-141-3p and miR-216a-5p were increased beyond vehicle treated dogs and did not display consistent time dependent increases. MiR-375-3p did display increases as compared to vehicle reaching its maximum increase at 1 h and remaining elevated until 24 h. In 15 μg/kg treated dogs miRs-216a-5p, 216b-5p, miR-375-3p and 141-3p were increased in a time dependent manner with maximal increases at 4–8 h (Fig. 9 and statistical analysis Additional file 16: Table S8). MiR-216a-5p displayed the greatest dynamic range reaching as high as 103 fold above vehicle. In the 45 μg/kg treated dogs amylase, lipase and miRNAs displayed dose and time dependent increases. MiR-216a-5p was increased maximally at 4–8 h and remained elevated until 72 h while levels as high as 5,494 fold above vehicle were observed in individual animals. MiR-216b-5p reached levels as high as 649 fold above vehicle in individual animals and remained elevated until 24 h. The remaining miRNAs did increase and remained elevated generally as long, but not to the degree of miRs-216a-5p and 216b-5p. Interestingly, liver enriched miR-122-5p was increased in a dose and time dependent manner while AST and ALT were increased in the 45 μg/kg treated dogs while no histopatholgic correlate was observed (data not shown). The rat caerulein study did not generate similar miR-122 increases. As demonstrated by these studies, miRNAs can serve as species translatable serum based biomarkers of pancreatic injury.

## Discussion

We have generated a rat miRNA body atlas and identified an additional ~1200 potential miRNAs in the rat as compared to the current number of rat miRNAs in miRBase v21. We have also confirmed many previously identified tissue specific and enriched miRNAs and have discovered many previously unknown rat tissue specific and enriched miRNAs. Rat and dog miRNA atlases were compared in order to determine which miRNAs are conserved and may represent species translatable biomarkers of organ injury. Subsequently, the conserved pancreas enriched miRNAs in rats and dogs were demonstrated in pancreas toxicology studies to be sensitive blood-based biomarkers of caerulein induced pancreatic injury in both species.

The aims of this study were 3 fold. First we sought to comprehensively catalogue the miRNA and isomiR content of the major organs of toxicologic interest in the rat in order to discover potential miRNA biomarkers of organ injury. Secondly, we wanted to identify tissue specific and tissue enriched miRNAs that may serve as biomarkers of organ injury. Thirdly, we wanted to test tissue specific and/or enriched miRNAs that are conserved in the rat and dog in toxicology studies to determine whether conserved miRNAs could be used as serum based biomarkers of organ injury in both species. While others have generated similar data using microarray or qPCR, next generation sequencing is advantageous in that it allows for the discovery of miRNA sequences without the need for prior genome annotation [41]. Analysis of the sequencing data refined the tissue specific/enrichment of previously reported tissue specific miRNAs and revealed many previously undiscovered tissue specific and enriched miRNAs.

### Identification of tissue specific and enriched miRs and isomiRs

Independent data analysis, utilizing different techniques, was performed by members of HESI. Approximately 165 tissue enriched miRNAs were found to be in common between the data analysis methods and additional tissue enriched miRNAs were identified by each institution. In order to ensure that interpretation of serum miRNA profiles is reflective of organ specific toxicity we sought to determine the tissue specificity and enrichment of the isomiRs of tissue specific and enriched miRNAs identified by Eli Lilly. Eli Lilly identified many sequences that mapped to mature miRNAs which consisted of sequences that differed from the mature miRNA sequence. These sequences, termed isomiRs [26] contained additional bases on the 5′ and 3′ ends as compared to the reference miRNA sequences and were also evaluated for tissue specificity and enrichment. We determined that the isomiRs of tissue specific and enriched miRNAs, with some exceptions, did generally mirror the expression of the reference miRNA sequence indicating that serum miRNA profiles should accurately reflect organ toxicity. Some isomiRs did display increased tissue specificity/enrichment as compared to the parent miRNA and these could serve as more specific markers of organ injury when faced with multiple organ toxicity.

Eli Lilly’s definition of a tissue enriched miRNA was purposefully vague because some miRNAs may be expressed in multiple organs at a higher level than the remaining organs, but may still be reasonable candidate biomarkers of organ injury depending on the observed toxicity. It is possible that a miRNA that is enriched in several tissues could be a useful marker of tissue injury if toxicity is restricted to a single organ that the miRNA is expressed in. In the face of multiple organ toxicity this approach may not be reasonable unless additional biomarkers could improve interpretation of the injury. Consequently, some miRNAs may not appear to be particularly tissue enriched as shown in Additional file 5: Figure S1.

### Rat and dog Pancreas Toxicity Studies

In order to characterize pancreas enriched miRNAs conserved between rat and dog as potential biomarkers of pancreatic injury we conducted rat and dog caerulein studies in which animals were treated with caerulein and concurrent clinical chemistry and histopathology were assessed along with miRNA changes in time course studies. Amylase and lipase increases were noted from 1–8 h in rats in both 15 and 50 μg/kg dose groups while pancreatic necrosis was noted at 8, 24 and 48 h. MiR-375-3p has been reported to be enriched in islets and the miRNA with the highest intra-islet expression [38] and in our study was increased from 4–24 h in the 15 and 50 μg/kg groups, returning to approximately vehicle level by 48 h. MiR-216a-5p and miR-217-5p remained elevated in the serum of rats longer than amylase or lipase and had a much greater dynamic range which could be advantageous if detection of pancreatic injury is not able to be examined at earlier time points.

Dogs treated with caerulein displayed serum amylase and lipase increases in the 15 μg/kg group from 1–8 h while showing increases in the 50 μg/kg group from 1–24 h. MiR-216a-5p levels increased prior to amylase and lipase and correlated well to amylase and lipase in the 15 and 50 μg/kg groups, but remained elevated longer and to a much larger degree than amylase and lipase. MiR-216b displayed similar kinetics to miR-216a-5p, but with a reduced dynamic range while miR-217-5p displayed increases similar to amylase. Interestingly, miR-217-5p displayed very large increases for the longest amount of time in the rat and this result was not reflected in the dog. This difference could reflect species differences in the toxicity of the compound, differences in the relative amount of the miRNA in dog pancreas or differences in the stability or clearance of the miRNA in dog serum. MiRs-141-3p and 200c-3p appeared to increase prior to amylase or lipase in the 3 μg/kg group and displayed increases similar to amylase in the 15 and 45 μg/kg groups while miR-148a-3p displayed increases similar to amylase. MiR-375-3p was increased prior to amylase and lipase at 3 μg/kg, correlated well with amylase and lipase in the 15 and 45 μg/kg dose groups and had a much larger dynamic range in the dog study as compared to the rat study. The other conserved pancreas enriched microRNAs tested were not correlated to clinical chemistry or histopathologic changes. These observations are important and demonstrate that miRNAs may display different kinetics in different species. Therefore, different miRNAs may have more favorable characteristics as biomarkers in different species and should be thoroughly investigated in each.

Interestingly, liver enriched miR-122-5p was increased in a dose and time dependent manner while AST and ALT were increased in the 45ug/kg treated dogs with no observed histopatholgic correlate. The rat caerulein study did not generate similar miR-122 increases. It is possible that, due to species differences in injury and physical proximity, pancreatic damage induced by caerulein in dogs caused some perturbation of the membranes of hepatocytes resulting in AST, ALT and miR-122-5p increases in the serum while not producing similar results in the rat. Additionally, individual rats and dogs displayed large differences in the amount of miRNA increased in the serum. This may be due to individual animal variation in the pancreatic damage, differing stability of the miRNAs or lack of miRNA normalization similar to tubulin or GAPDH used for mRNA comparisons as all samples were normalized by serum volume.

We tested several conserved pancreas enriched miRNAs in rat and dog pancreas toxicity studies and determined that the miRNAs perform as well or better than traditional markers of pancreas injury. Based on these studies miR-216a-5p displayed the largest dynamic range and correlated well with amylase and lipase indicating that it is the best translatable miRNA biomarker that correlated to pancreatic injury from rat to dog. It is possible that additional pancreas specific and/or enriched miRNAs may supplement the interpretation of pancreatic injury or serve as better biomarkers than miR-216a-5p in pancreatic toxicity studies utilizing different pancreatic toxicants. The data generated in this study verified our rationale for generating a library of rat tissue/organ specific and enriched miRNAs. These data can serve as a resource for future research by the scientific community for discovery and characterization of additional miRNA biomarkers as well as a resource for more comprehensive isomiR analysis.

### Additional considerations

While the work here has advanced the knowledge of the miRNA and isomiR content of rat organs it is important to note that the quantitative capacity of miRNA sequencing may be inaccurate due to ligase bias as certain miRNAs may be under or over-represented and potentially not detected [42–44]. The sequencing data and subsequent analysis did identify many tissue enriched miRNAs previously discovered through other methods, therefore it is reasonable to infer that ligase bias may not be dependent on the RNA content of a particular sample, but may be dependent on the base composition of an individual miRNA. Ligase bias most certainly affected the quantitative capacity of miRNA sequencing in this study (Additional file 17: Figure S7 and Additional file 18: Figure S8) as the quantitation of miRs-216a-5p, 375-3p and 148a-3p did differ between the sequencing data and qPCR. It is also possible that ligase bias could affect quantitation of isomiRs and this could affect which miRNA sequences are targeted for detection in the serum by qPCR or other technologies. QPCR assays in the caerulein studies conducted here were chosen based upon the sequences that aligned to the mature miRNA with the highest expression level. This may have resulted in picking an isomiR sequence that was expressed at a different level than the sequencing data indicated. If a miRNA is not expressed at a high level in a tissue then it would be reasonable to conclude that little would be detectable in the serum and this could be an additional reason why some miRNAs did not appear to perform as well as others in the toxicology studies conducted here. In addition, as methods for improving ligase bias improve, reanalysis of the miRNA content of rat organs may need to be performed or analytical models may be developed that could account for ligase bias to gain a true understanding of miRNA quantity in each organ and potentially detect additional miRNAs. IsomiRs generally displayed more variation on the 3′ ends which can affect the selection of the most appropriate qPCR assay to use for detection, therefore selection of the sequence present in the greatest amount should result in optimal detection of injury if the miRNA is released upon cellular injury, stable in the blood, present in sufficient amount and ligase bias is not a confounding factor. Improvements in the quantitative capacity of miRNA sequencing could also result in changing which miRNA is defined as the mature miRNA sequence in miRBase and other miRNA cataloguing databases.

An advantage of miRNAs as biomarkers of organ injury is the potential for species translatability of the biomarker for organ toxicity using the same assay to detect the miRNA. It is possible that expression of miRs and isomiRs could change in the tissues of compound treated animals. Therefore, it would be worthwhile to determine if the tissue specificity/enrichment and expression levels of miRNA sequences change in response to toxic insult. Ultimately, toxicology studies examining the kinetics of tissue specific and enriched miRNAs utilizing a variety of compounds eliciting toxicity in a particular organ in different species will likely be needed to establish which microRNAs are the best species translatable serum based biomarkers of organ injury. Studies may also need to be conducted with a wide variety of toxicants to determine which miRNAs are subject to the least amount of change in expression level in serum in order to determine if a serum miRNA could be used for normalization. Additionally, determining reference ranges for miRNAs of interest will be a topic that will need to be investigated with additional toxicology studies in the future. This could assist researchers in determining what degree of change correlates to what degree of organ injury. Clearly, miRNAs will need to be rigorously evaluated in a manner similar to other biomarkers [45, 46]. While the major organs of toxicologic interest were examined, not all tissues in the rat or dog were examined. Therefore, tissues such as the thyroid, parathyroid, lung, spleen, fat, bone, cartilage, skin, spinal cord, peripheral neurons and other tissues could also be examined in future studies for their miRNA content. Moreover, as shown in this study, different data analysis methods can generate different numbers of tissue enriched miRNAs. These data may yield additional candidate biomarkers of organ injury if different data analysis methods are employed.

As further characterization of serum miRNA biomarkers is conducted, a qPCR based panel or a method utilizing multiplexing of miRNAs representative of each organ with multiple miRNAs per organ may be generated and used regularly in toxicology studies utilizing biomarkers as the major or a complimentary method for determining organ toxicity. This method could ultimately detect toxicity in organs with validated miRNA biomarkers using a small volume of serum and may significantly reduce the cost of preclinical and clinical drug development.

## Conclusions

Using a next generation sequencing approach, we have generated a rat miRNA body atlas. In that process we discovered an additional 1,162 potential rat miRNAs, verified previously identified tissue specific and enriched miRNAs, identified many new tissue specific and enriched miRNAs, and analyzed isomiR expression of tissue specific and enriched miRNAs. In addition, we provided proof of concept that pancreas enriched miRNAs conserved between rat and dog could be used as serum based markers of pancreatic injury in rat and dog caerulein toxicology studies. In collaboration with Takeda and HESI we have analyzed the dog miRNA sequencing data and determined which tissue specific and enriched miRNAs were conserved between the 2 species. Finally, we determined that miR-216a-5p appears to perform as well or better as a marker of pancreatic injury than amylase, lipase or other pancreas enriched miRNAs in rat and dog caerulein toxicity studies.

## Additional files

Additional file 1: Supplementary Methods. Eli Lilly miRDeep 2 novel miRNA identification (DOCX 13 kb) Additional file 2: Sequences of miRNAs and Exiqon qPCR assay identification. (XLSX 14 kb) Additional file 3: Table S1. List of miRNAs in common in HESI analysis. Tissue enriched miRNAs mutually identified by independent data analysis techniques employed by Eli Lilly, NIEHS and Maastricht University are listed including tissue (s) in which the miRNA is most abundantly expressed. Sequences of miRNAs and Exiqon qPCR assay identification. (XLSX 12 kb) Additional file 4: Table S2. Tissue specific and enriched miRNAs identified by Lilly. Tissue specific and tissue enriched miRNAs identified by Eli Lilly are listed under the organ in which they are most abundantly expressed. Tissue specific miRNAs are in bold text and tissue enriched miRNAs are in non-bold text. (XLSX 21 kb) Additional file 5: Figure S1. Tissue specific and enriched miRNAs identified by Lilly. Tissue specific and enriched miRNAs identified by Eli Lilly are displayed in a heat map with tissues listed on the X-axis and miRNAs listed on the Y-axis. Expression levels are indicated by a range of colors with blue indicating low expression and red indicating high expression. *P*-values are included next to each miRNA indicating the degree of tissue specificity and/or enrichment. Some miRNAs may be enriched in several tissues resulting in a high *p*-value. (PDF 88 kb) Additional file 6: Table S3. Tissue Enriched miRNAs identified by NIEHS. Tissue enriched miRNAs identified by NIEHS are listed under the organ in which they are most abundantly expressed. (XLSX 42 kb) Additional file 7: Table S4. Tissue Enriched miRNAs identified by Maastricht University. Tissue enriched miRNAs identified by Maastricht University are listed under the organ in which they are most abundantly expressed. (XLSX 18 kb) Additional file 8: Table S5. MiRNAs tested in qPCR validation. Tissue enriched miRNAs and their sequences are displayed. MiRNA abundance was measured through qPCR in order to correlate their tissue enrichment status to deep sequencing results. (XLSX 15 kb) Additional file 9: Table S6. Correlations of miRNAs to sequencing data. Correlation of tissue enriched miRNA measurements using deep sequencing and qPCR are depicted with a correlation of −1.0 being highly correlated and −0.0 representing poor correlation. (XLSX 10 kb) Additional file 10: Figure S2. IsomiRs of pancreas enriched miRNAs. The isomiRs of pancreas enriched miRNAs and their sequences are displayed in a heat map with tissues listed on the X-axis and isomiRs listed on the Y-axis. Expression levels are indicated by a range of colors with blue indicating low expression and red indicating high expression. IsomiRs of miR-217-5p display more expanded or restricted expression with respect to tissues. (PDF 17 kb) Additional file 11: Figure S3. IsomiRs of miR-215 in the intestine. The isomiRs of select intestine enriched miRNAs and their sequences are displayed in a heat map with tissues listed on the X-axis and isomiRs listed on the Y-axis. Expression levels are indicated by a range of colors with blue indicating low expression and red indicating high expression. IsomiRs of miR-215 display more expanded or restricted expression with respect to tissues. (PDF 13 kb) Additional file 12: Figure S4. IsomiRs of miR-192-5p in the liver. The isomiRs of select liver enriched miRNAs and their sequences are displayed in a heat map with tissues listed on the X-axis and isomiRs listed on the Y-axis. Expression levels are indicated by a range of colors with blue indicating low expression and red indicating high expression. IsomiRs of miR-192-5p display more expanded or restricted expression with respect to tissues. (PDF 14 kb) Additional file 13: Figure S5. miR-3473f-pre isomiRs in the testis. The isomiRs of select testis enriched miRNAs and their sequences are displayed in a heat map with tissues listed on the X-axis and isomiRs listed on the Y-axis. Expression levels are indicated by a range of colors with blue indicating low expression and red indicating high expression. IsomiRs of miR-3473f-pre display more expanded or restricted expression with respect to tissues. (PDF 14 kb) Additional file 14: Figure S6. miR-3473f-pre isomiRs in the testis. The isomiRs of select testis enriched miRNAs and their sequences are displayed in a heat map with tissues listed on the X-axis and isomiRs listed on the Y-axis. Expression levels are indicated by a range of colors with blue indicating low expression and red indicating high expression. IsomiRs of miR-3473f-pre display more expanded or restricted expression with respect to tissues. (PDF 14 kb) Additional file 15: Table S7. P-values of rat caerulein toxicity study. Statistical analysis of miRNA and clinical chemistry biomarkers measured in the rat caerulein toxicity study are presented in this table with their associated fold changes and *p*-values indicated at 1 h (yellow), 4 h (green), 8 h (blue), 24 h (orange) and 48 h (gray). (XLSX 13 kb) Additional file 16: Table S8.
*P*-values of dog caerulein toxicity study. Statistical analysis of miRNA biomarkers measured in the dog caerulein toxicity study are displayed in this table with their associated p-values, Lsmean, lower confidence intervals, upper confidence intervals and t ratios indicated at 1 h (yellow), 4 h (green), 8 h (blue), 24 h (orange) and 48 h (gray) for the each dose group. (XLSX 27 kb) Additional file 17: Figure S7. Representation of rat miRNA sequencing data. Rat miRNA sequencing data is represented with the normalized reads per million on the Y-axis and the tissues on the X-axis. Each color within the figure represents an individual miRNA while the height of the colored bar represents the percentage of reads that a particular miRNA makes up within that tissue. MiRs-216a-5p, 375-3p and 148a-3p are indicated in the yellow, green and pink bars in the pancreas tissue respectively. (PDF 41 kb) Additional file 18: Figure S8. qPCR of pancreas enriched miRNAs. Total RNA from the pancreas tissue of each individual rat was normalized by mass and examined by qPCR for expression of miRs-216a-5p, 375-3p and 148a-3p. Ct values are indicated on the Y-axis and the rat pancreas samples are indicated on the X-axis. Each miRNA appears to be expressed at approximately the same Ct value indicating they are present at roughly the same quantity in the pancreas. (PDF 32 kb)

## Abbreviations

ALP: alkaline phosphatase
AST: aspartate aminotransferase
ALT: alanine aminotransferase
BUN: Blood urea nitrogen
CNS: central nervous system
CO_2_: carbon dioxoide
Creatinine: urinary albumin
KIM-1: kidney injury molecule-1
cTnI: Cardiac troponin I
GGT: gamma-glutamyl transferase
GLDH: glutamate dehydrogenase
HESI: Health and Environmental Sciences Institute
HMGB1: high mobility group protein B1
miR: microRNA
NGAL: neutrophil gelatinase associated lipocalin
qPCR: quantitative polymerase chain reaction
RISC: RNA Induced silencing complex
RNA: ribonucleic acid
SD: Sprague Dawley
TMPD: 2,3,5,6 tetramethyl-p-phenylenediamine

## Footnotes

1 This research was performed in collaboration with HESI Genomics Committee

## References

[1] Van Alain J, Gool BH (2010). From biomarker strategies to biomarker activities and back. Drug Discov Today.

[2] Mona Botros KAS (2013). The De Ritis Ratio: The test of time. Clin Biochem Rev.

[3] Josef Ozer MR, Martin S, Wendy B, Shelli S (2008). The current state of serum biomarkers of hepatotoxicity. Toxicology.

[4] Boon A, Boon ARaG (1983). A case oeiented approach to small animal biochemical profiling.

[5] Amacher DE (1997). Serum transaminase elevations as indicators of hepatic injury following the administration of drugs. Regul Toxicol Pharmacol.

[6] L. Boone DM, Cusick P, Ennulat D, Provencher Bolliger A, Everds N, Meador V, Elliott G, Honor D, Bounous D, Jordan H (2005). Selection and interpretation of clinical pathology indicators of hepatic injury in preclinical studies. Vet Clin Pathol.

[7] Engle SK, Pritt ML, Chiang AY, Davis MA, Zimmerman JL, Rudmann DF, Kathleen H-T, Irrizarry AR, Yumi Y, David Mendel A, Eric S, Cornwell PD, Watson DE (2009). Qualification of Cardiac troponin I concentrration in mouse serum using isoproterenol and implementation in pharmacology studies to accelerate drug development. Toxicol Pathol.

[8] Dieterle PY (2009). Tissue-specific, non-invasive toxicity biomarkers: translation from preclinical safety assessment to clinical safety monitoring. Expert Opin Drug Metab Toxicol.

[9] Olivier Le Moine H, Jaques D, Philippe T, Michel C, Hendi-Albert O (1994). Trypsin Activity A New Marker of Acute Alcoholic Pancreatitis. Dig Dis Sci.

[10] Harry Olson GB, Denise R, Karluss T, Alastair M, Gerald K, Patrick L, James S, Glenn S, William B, Michael D, Koen Van D, Peter S, Bruce B, Allen H (2000). Concordance of the Toxicity of Pharmaceuticals in Humans and in Animals. Regul Toxicol Pharmacol.

[11] Chihiro Tamaki TN, Masamichi H, Masato F, Masanori H, Hiroshi K, Minoru N, Kazuhiko S, Yoshiharu T, Yamato O, Daisaku Y, Yasuo Y, Shigeru H, Takako O, Kazuichi N (2013). Potentials and limitations of non clinical safety assessment for predicting clinical adverse drug reactions: correlation analysis of 142 approved drugs in Japan. J Toxicol Sci.

[12] Bartel DP (2004). MicroRNAs: Genomics, Biogenesis, Mechanism, and Function. Cell.

[13] Jessica A, Weber DHB, Shile Z, David Y, Huang K, How H, Ming Jen L, Galas DJ, Kai W (2010). The MicroRNA Spectrum in 12 Body Fluids. Clin Chem.

[14] Mestdagh P, S.L., Pattyn F, Ridzon D, Fredlund E, Fieuw A, Ongenaert M, Vermeulen J, De Paepe A, Wong L, Speleman F, Chen C, Vandesompele J. The microRNA body map: dissecting microRNA function through integrative genomics. Nucleic Acids Res. 2011;39(20):1–8. 10.1093/nar/gkr646 PMC3203610 21835775

[15] Sam EV Linsen E, De Bruijn E, Edwin C (2010). Small RNA expression and strain specificity in therat. BMC Genomics.

[16] Mariana Lagos-Quintana RR, Abdullah Y, Jutta M, Winfried L, Thomas T (2002). Identification of Tissue-Specific MicroRNAs from Mouse. Curr Biol.

[17] Keiichi Minami TU, Yuji M, Ko O, Masayuki K, Akira H, Atsushi O, Hiroshi Y, Yasuo O, Tetsuro U (2013). miRNA expression atlas in male rat. Nature/scientific data.

[18] Patrick S, Mitchell RKP, Kroh EM, Fritz BR, Wyman SK, Pogosova-Agadjanyan EL, Amelia P, Jennifer N, O’Briant KC, April A, Lin DW, Nicole U, Drescher CW, Knudsen BS, Stirewalt DL, Robert G, Vessella RL, Nelson PS, Martin DB, Muneesh T (2008). Circulating microRNAs as stable blood-based markers for cancer detection. Proc Natl Acad Sci.

[19] Keiichi Minami TU, Yuji M, Ko O, Masayuki K, Akira H, Atsushi O, Hiroshi Y, Yasuo O, Tetsuro U (2014). miRNA expression atlas in male rat. Scientific Data.

[20] Mariana Lagos-Quintana RR, Winfried L, Thomas T (2001). Identification of Novel Genes Coding for Small Expressed RNAs. Science.

[21] Julien Roux MG (2012). Comparative analysis of human and mouse expression data illuminates tissue-specific evolutionary patterns of miRNAs. Nucleic Acids Res.

[22] Laterza OF, Garrett-Engele PW, Katerina V, Nagaraja M, Tanaka WK, Johnson JM, Sina JF, Fare TL, Sistare FD, Glaab WE (2009). Plasma MicroRNAs as Sensitive and Specific Biomarkers of Tissue Injury. Clin Chem.

[23] Kai Wang SZ, Bruz M, Pamela T, Amy B, Zhiyuan H, Hood LE, Galas DJ (2009). Circulating microRNAs, potential biomarkers for drug-induced liver injury. Proc Natl Acad Sci.

[24] Starkey PJ, Lewis JD, Vivien P, Simpson KJ, Darren GN C, Antoine DJ, French NS, Neeraj D, Webb DJ, Costello EM, Neoptolemos JP, Jonathan M, Goldring CE, Kevin Park B (2011). Circulating MicroRNAs as Potential Markers of Human Drug-Induced Liver Injury. Hepatology.

[25] Daniel J, Antoine JWD, Philip Starkey L, Vivien P, Judy C, Moyra M, Thanacoody RH, Gray AJ, Webb DJ, Moggs JG, Nicholas Bateman D, Goldring CE, Kevin Park B (2013). Mechanistic Biomarkers Provide Early and Sensitive Detection of Acetaminophen-Induced Acute Liver Injury at First Presentation to Hospital. Hepatology.

[26] Ryan D, Morin MDOC, Malachi G, Florian K, Allen D, Anna-Liisa P, Yongjun Z, Helen MD, Thomas Z, Martin H, Eaves CJ, Marra MA (2008). Application of massively parallel sequencing to microRNA profiling and discovery in human embryonic stem cells. Genome Res.

[27] Covaris Tissue Pulverization Protocol

[28] Martin M (2012). Cutadapt removes adapter sequences from high-throughput sequencing reads. Bioinformatics in Action.

[29] Marc R, Friedländer SDM, Na L, Wei C, Nikolaus R (2012). miRDeep2 accurately identifies known and hundreds of novel microRNA genes in seven animal clades. Nucl Acids Res.

[30] Weixiong Zhang SG, Xuefeng Z, Jing X, Padmanabhan C, Xiang Z, Xiaoming Z, Hailing J (2010). Multiple distinct small RNAs originate from the same microRNA precursors. Genome Biol.

[31] Jean-Philippe Brunet PT, Golub TR, Mesirov JP (2004). Metagenes and molecular pattern discovery using matrix factorization. PNAS.

[32] Friedländer MR, Catherine Adamidi WC, Jonas M, Ralf E, Signe K, Nikolaus R (2008). Discovering microRNAs from deep sequencing data using miRDeep. Nat Biotechnol.

[33] Kay Prüfer US, Michael D, Green RE, Michael L, Janet K (2008). PatMaN: rapid alignment of short sequences to large databases. Bioinformatics.

[34] Steven P, Lund DN, McCarthy DJ, Smyth GK (2012). Detecting Differential Expression in RNA-sequence Data Using Quasi-likelihood with Shrunken Dispersion Estimates. Stat Appl Genet Mol Biol.

[35] Goro K, Keiko Imoda HO, Kenji M (1994). Combined high performance liquid chromatography and radioimmunoassay for cerulitide and its metabolites in dog plasma and urine. J Pharm Biomed Anal.

[36] Li Y, Liu F, Z.Z., Vongsangnak W, Jing Q, Shen B. Performance comparison and evaluation of software tools for microRNA deep-sequencing data analysis. Nucleic Acids Res. 2012;1–8. doi:10.1093/nar/gks043. 10.1093/nar/gks043 PMC3378883 22287634

[37] Allmer MY (2014). miRNomics MicroRNA Biology and Computational Analysis. Methods Mol Biol.

[38] Valia Bravo-Egana SR, Damaris Molano R, Antonello P, Camillo R, Juan D’n-B, Pastori RL (2008). Quantitative differential expression analysis reveals miR-7 as major islet microRNA. Biochem Biophys Res Commun.

[39] Xiang-Yu K, QD Y, Lei L, Jian-Qiang L, Guo-Kun W, Jia-Qi Z, Xiao-Hua M, Yan-Fang G, Li-Ning X, Yong-Zhi Z, Shang-Xin D, Jun-Jun G, Zhao-Shen L (2010). Plasma miR-216a as a potential marker of pancreatic injury in a rat model of acute pancreatitis. World J Gastroenterol.

[40] Guo-Kun W, QZ J, Jun-Tao Z, Qing L, Yue L, Jia H, Yong-Wen Q, Qing J (2010). Circulating microRNA: a novel potential biomarker for early diagnosis of acute myocardial infarction in humans. Eur Heart J.

[41] Lik Wee L, Alton Etheridge SZ, Li M, Dan M, David G, Kai W (2010). Complexity of the microRNA repertoire revealed by next-generation sequencing. RNA.

[42] Karim Sorefan HP, Hall AE, Ana K, Sam G-J, Vincent M, Dalmay T (2012). Reducing ligation bias of small RNAs in libraries for next generation sequencing. Silence.

[43] Zhaojie Zhang JEL, Kent R, Anderson EM, Rui Y (2013). High-efficiency RNA cloning enables accurate quantification of miRNA expression by deep sequencing. Genome Biol.

[44] Jayaprakash AD, O.J., Brown BD, Sachidanandam R. Identification and remediation of biases in the activity of RNA ligases in small-RNA deep sequencing. Nucleic Acids Res. 2011; 1–12. doi:10.1093/nar/gkr693 10.1093/nar/gkr693 PMC3241666 21890899

[45] Melanie Blank ADF, Federico G, Patricia H, Elizabeth H, David J-K, William T, Aliza T, Douglas T, Shen X (2009). Review of Qualification Data for Biomarkers of Nephrotoxicity Submitted by the Predictive Safety Testing Consortium. Center for Drug Evaluation and Research U.S. Food and Drug Administration.

[46] PJ O’Brien WR, York MJ, Jacobsen MC (2011). Review of Qualification Data for Cardiac Troponins.

