# Supplementary material for: The Rat microRNA body atlas; Evaluation of the microRNA content of rat organs through deep sequencing and characterization of pancreas enriched miRNAs as biomarkers of pancreatic toxicity in the rat and dog
Source: BMC Genomics. 2016 Aug 30;17(1):694. doi: 10.1186/s12864-016-2956-z (PMC5006322; doi:10.1186/s12864-016-2956-z)
Supplement: Additional file 1: — Supplementary Methods. Eli Lilly miRDeep 2 novel miRNA identification (DOCX 13 kb) [file 12864_2016_2956_MOESM1_ESM.docx]

Supplementary Methods

Eli Lilly miRDeep 2 novel miRNA identification

We aligned all the unique trimmed fasta reads to the RN5 reference rat genome with miRDeep2’s mapper.pl script with default parameters and then used the miRDeep2.pl script to generate novel miR predictions and .mrd files. The .mrd files were parsed as above to assign miR names to each unique read. We then attempted to identify and merge novel predictions that were identical to known miRs as follows. If the same read sequence identified a miR in the novel analysis and in the known analysis and the known miR appeared in miRDeep’s .mrd file, we assumed that this was a duplicate identification and changed the miR name from that reported by miRDeep (chr# followed by a unique ID) to that of the known miR. Otherwise, we assumed that the novel prediction might be different from the known miR identification and added it to the list of potential identifications for the sequence. So, for example, a sequence which was aligned by miRDeep2 to a predicted miR on chrX and also identified as miR-450a is identified as “chrX_48156-5p;miR-450a-5p” because miRDeep2 does not show miR-450a as aligned to this predicted miR indicating that these are two potentially distinct sources of this sequence. Finally, the counts associated with each unique sequence are summed for each named miR (including complex, multi-named miRs) for each sample and these are reported as the miR level counts.
