# Supplementary figures and images for: The Rat microRNA body atlas; Evaluation of the microRNA content of rat organs through deep sequencing and characterization of pancreas enriched miRNAs as biomarkers of pancreatic toxicity in the rat and dog
Source: BMC Genomics. 2016 Aug 30;17(1):694. doi: 10.1186/s12864-016-2956-z (PMC5006322; doi:10.1186/s12864-016-2956-z)

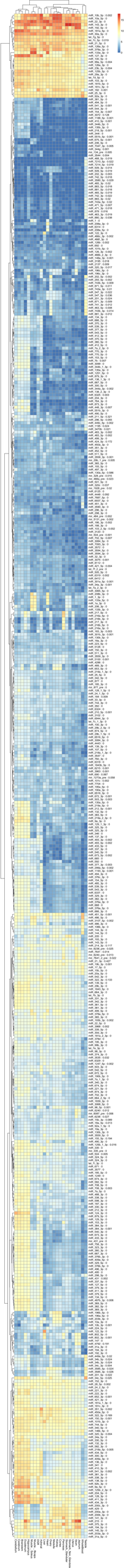

Supplement: Additional file 5: Figure S1. — Tissue specific and enriched miRNAs identified by Lilly. Tissue specific and enriched miRNAs identified by Eli Lilly are displayed in a heat map with tissues listed on the X-axis and miRNAs listed on the Y-axis. Expression levels are indicated by a range of colors with blue indicating low expression and red indicating high expression. P-values are included next to each miRNA indicating the degree of tissue specificity and/or enrichment. Some miRNAs may be enriched in several tissues resulting in a high p-value. (PDF 88 kb) [file 12864_2016_2956_MOESM5_ESM.pdf]

### Heatmap for Pancreas (counts on logscale)

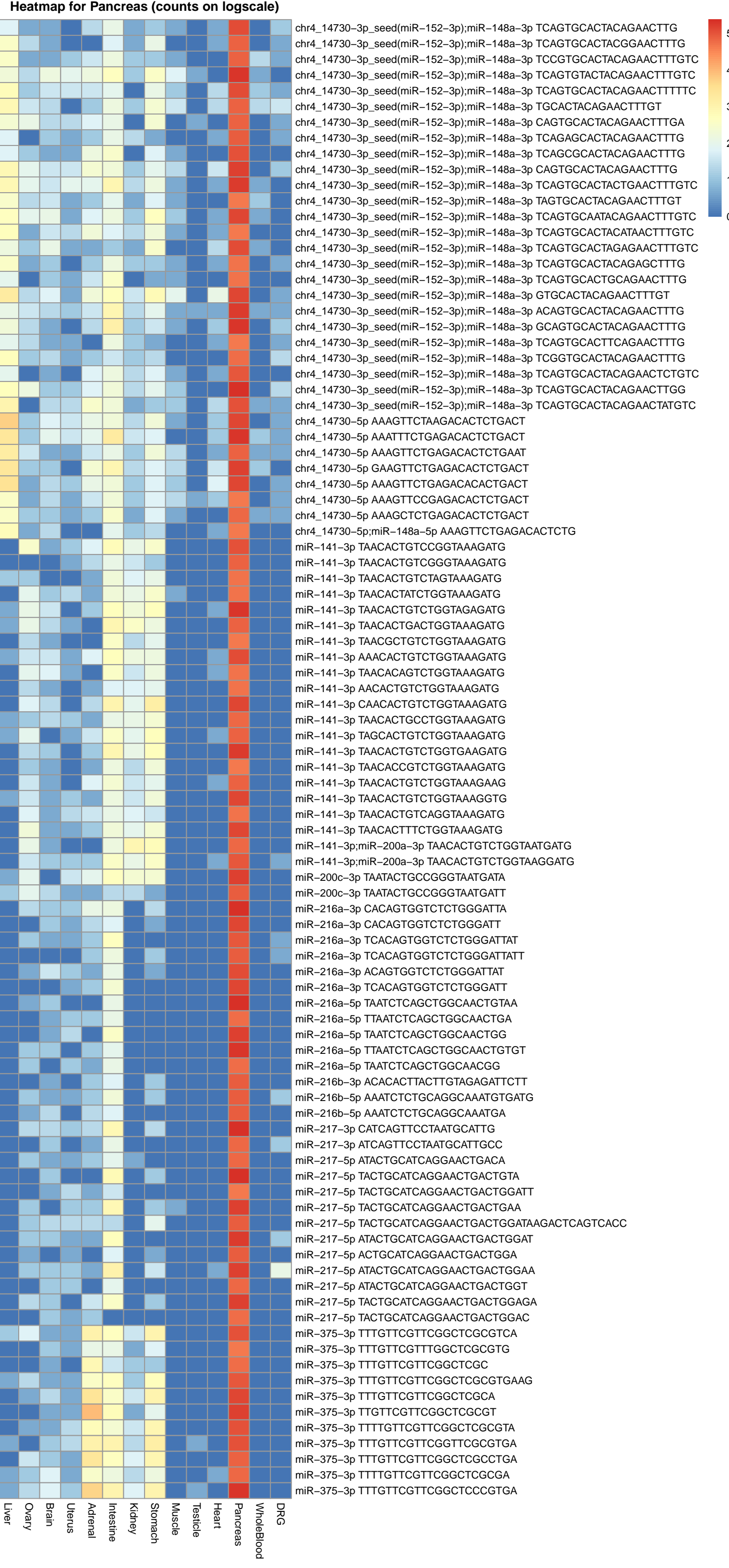

Supplement: Additional file 10: Figure S2. — IsomiRs of pancreas enriched miRNAs. The isomiRs of pancreas enriched miRNAs and their sequences are displayed in a heat map with tissues listed on the X-axis and isomiRs listed on the Y-axis. Expression levels are indicated by a range of colors with blue indicating low expression and red indicating high expression. IsomiRs of miR-217-5p display more expanded or restricted expression with respect to tissues. (PDF 17 kb) [file 12864_2016_2956_MOESM10_ESM.pdf]

Heatmap for Intestine (counts on logscale)

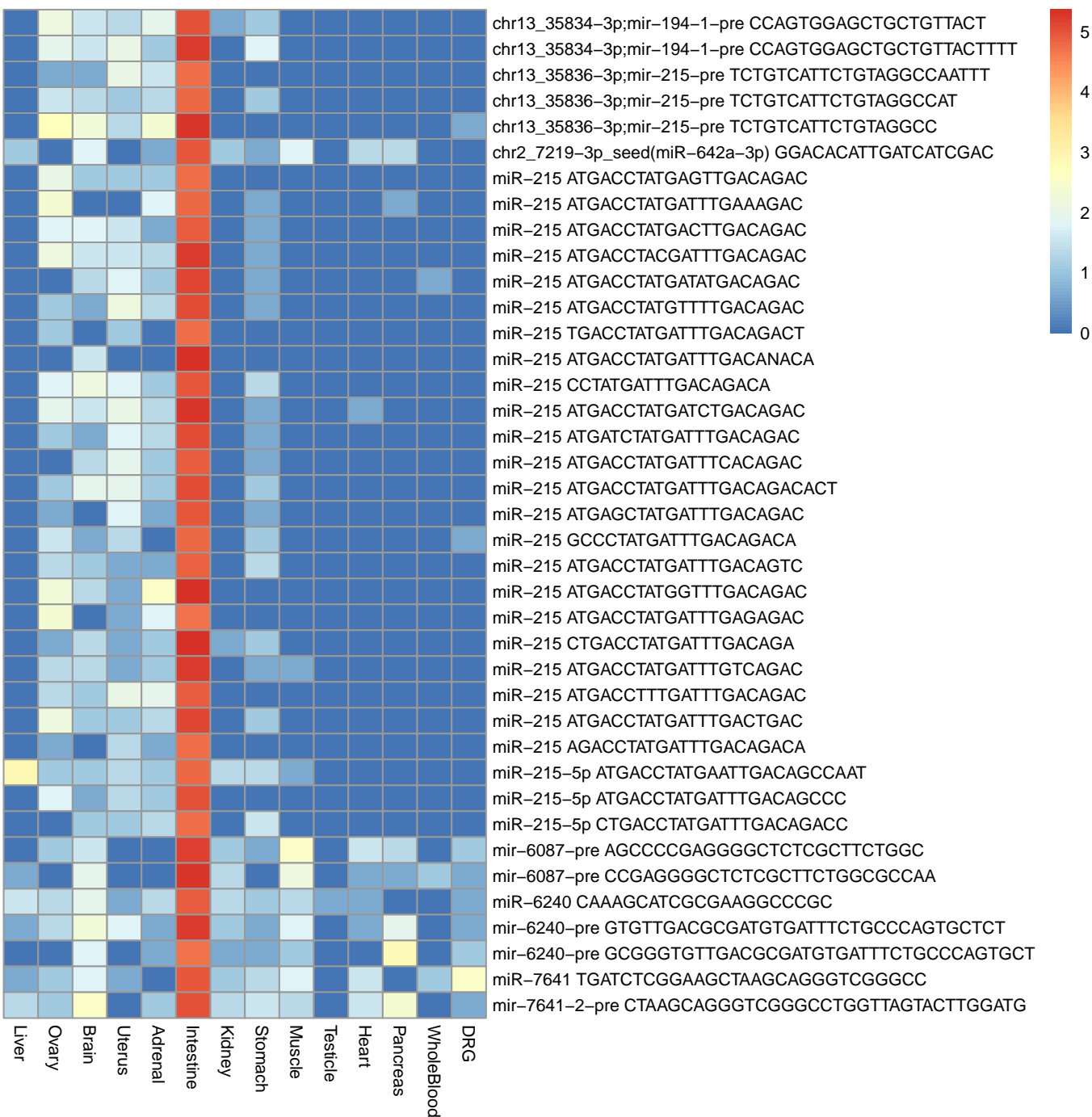

Supplement: Additional file 11: Figure S3. — IsomiRs of miR-215 in the intestine. The isomiRs of select intestine enriched miRNAs and their sequences are displayed in a heat map with tissues listed on the X-axis and isomiRs listed on the Y-axis. Expression levels are indicated by a range of colors with blue indicating low expression and red indicating high expression. IsomiRs of miR-215 display more expanded or restricted expression with respect to tissues. (PDF 13 kb) [file 12864_2016_2956_MOESM11_ESM.pdf]

Heatmap for Liver (counts on logscale)

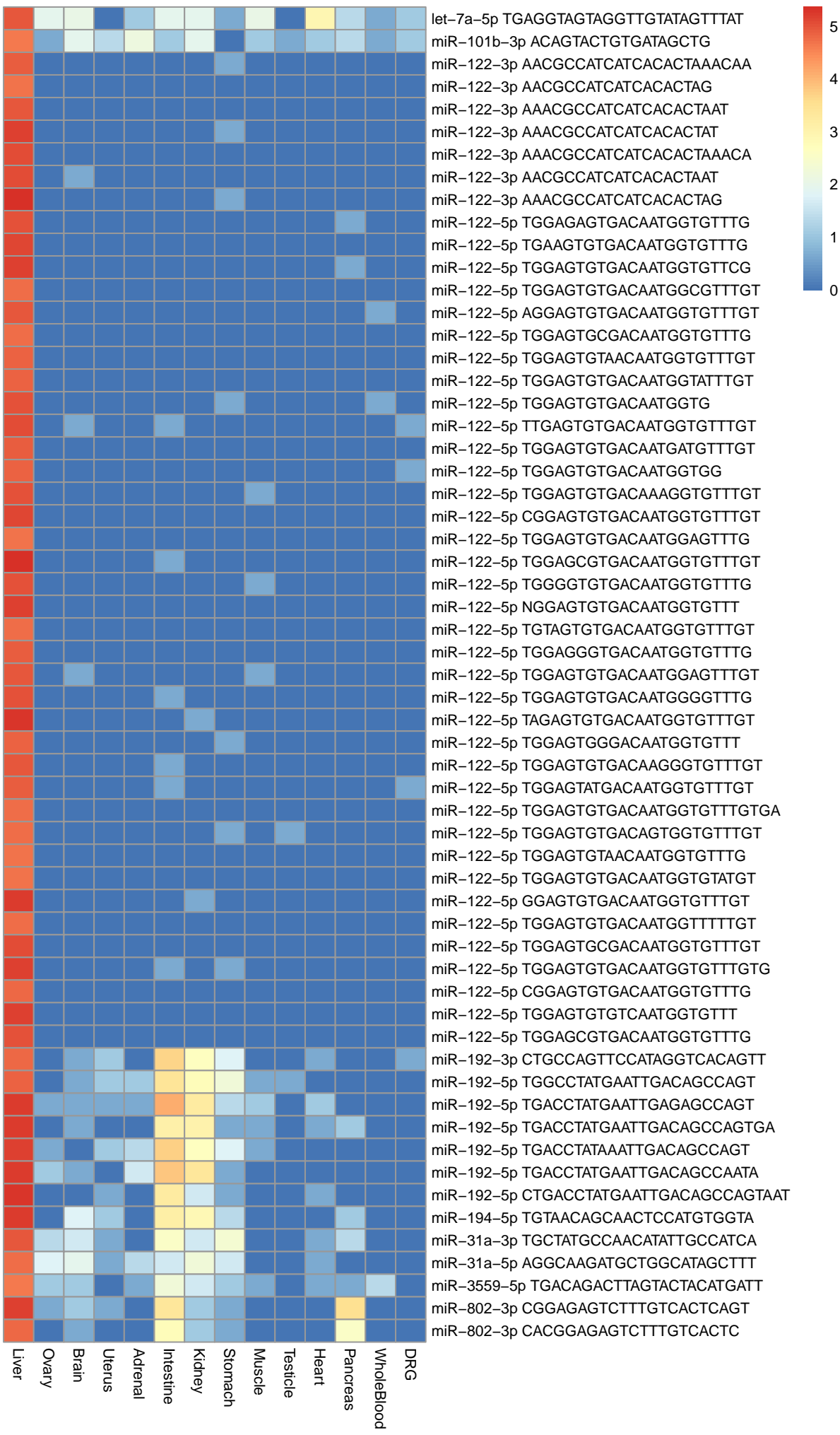

Supplement: Additional file 12: Figure S4. — IsomiRs of miR-192-5p in the liver. The isomiRs of select liver enriched miRNAs and their sequences are displayed in a heat map with tissues listed on the X-axis and isomiRs listed on the Y-axis. Expression levels are indicated by a range of colors with blue indicating low expression and red indicating high expression. IsomiRs of miR-192-5p display more expanded or restricted expression with respect to tissues. (PDF 14 kb) [file 12864_2016_2956_MOESM12_ESM.pdf]

Heatmap for Testicle (counts on logscale)

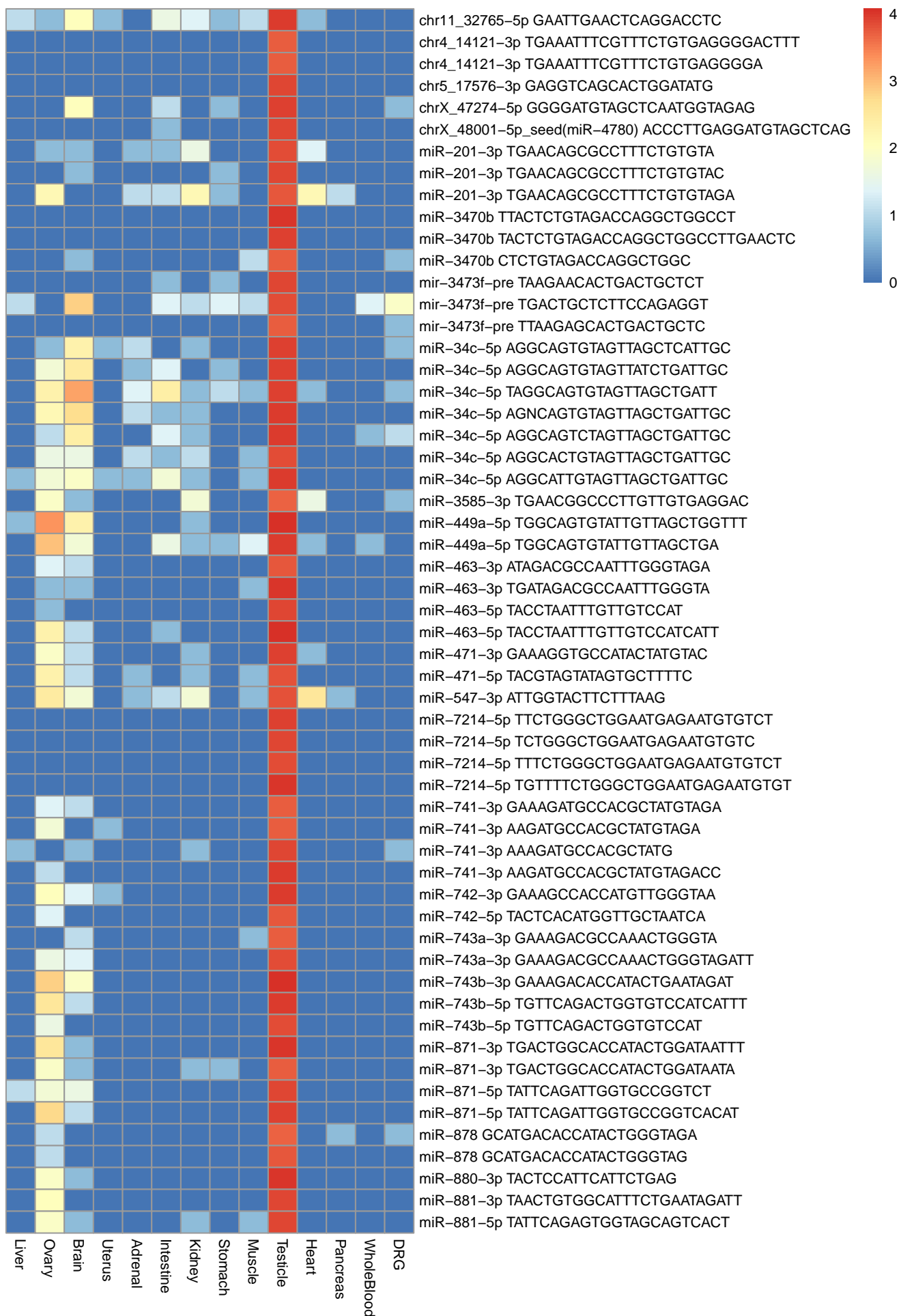

Supplement: Additional file 13: Figure S5. — miR-3473f-pre isomiRs in the testis. The isomiRs of select testis enriched miRNAs and their sequences are displayed in a heat map with tissues listed on the X-axis and isomiRs listed on the Y-axis. Expression levels are indicated by a range of colors with blue indicating low expression and red indicating high expression. IsomiRs of miR-3473f-pre display more expanded or restricted expression with respect to tissues. (PDF 14 kb) [file 12864_2016_2956_MOESM13_ESM.pdf]

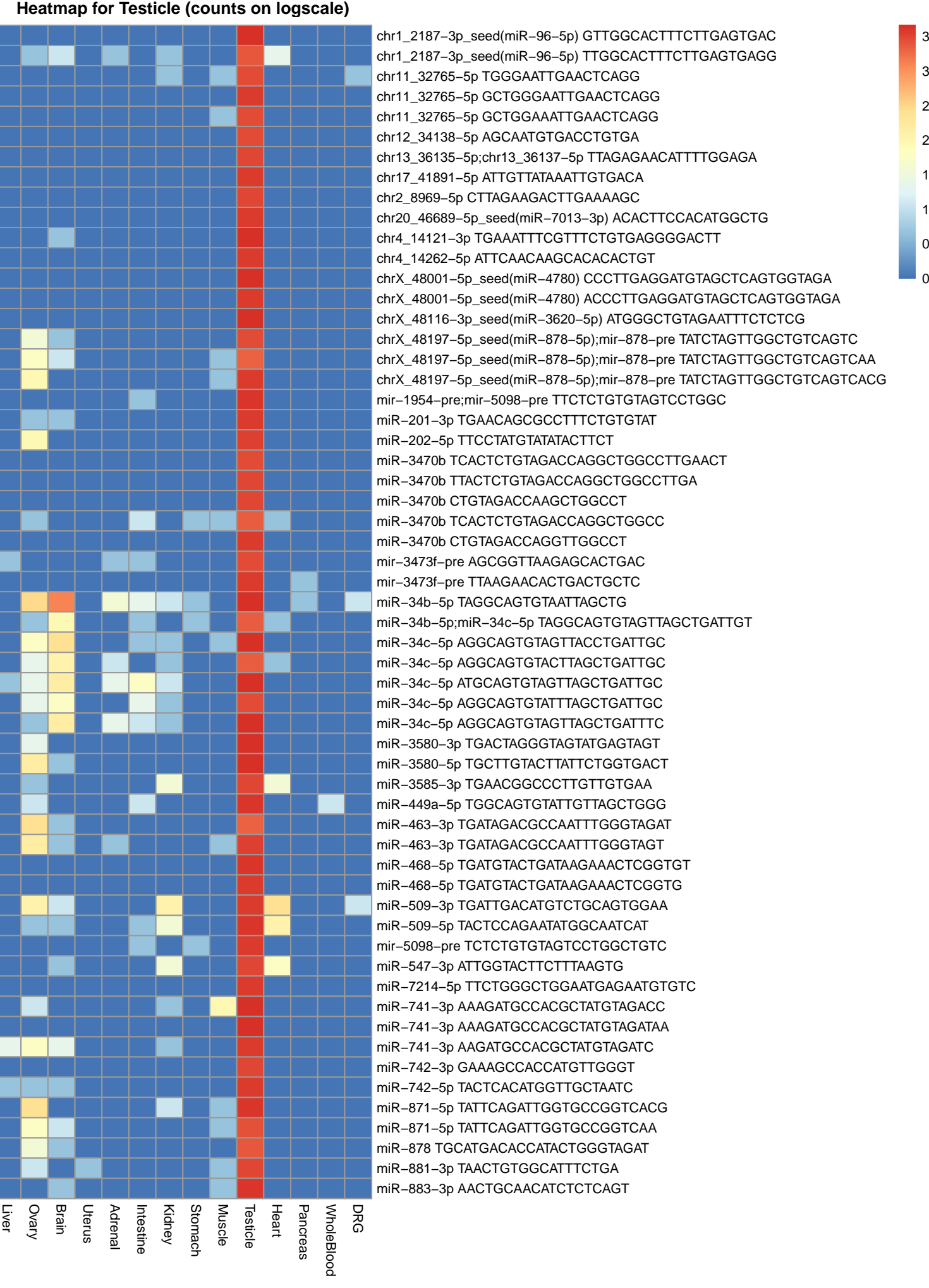

Supplement: Additional file 14: Figure S6. — miR-3473f-pre isomiRs in the testis. The isomiRs of select testis enriched miRNAs and their sequences are displayed in a heat map with tissues listed on the X-axis and isomiRs listed on the Y-axis. Expression levels are indicated by a range of colors with blue indicating low expression and red indicating high expression. IsomiRs of miR-3473f-pre display more expanded or restricted expression with respect to tissues. (PDF 14 kb) [file 12864_2016_2956_MOESM14_ESM.pdf]

# Rat miRNA body atlas data

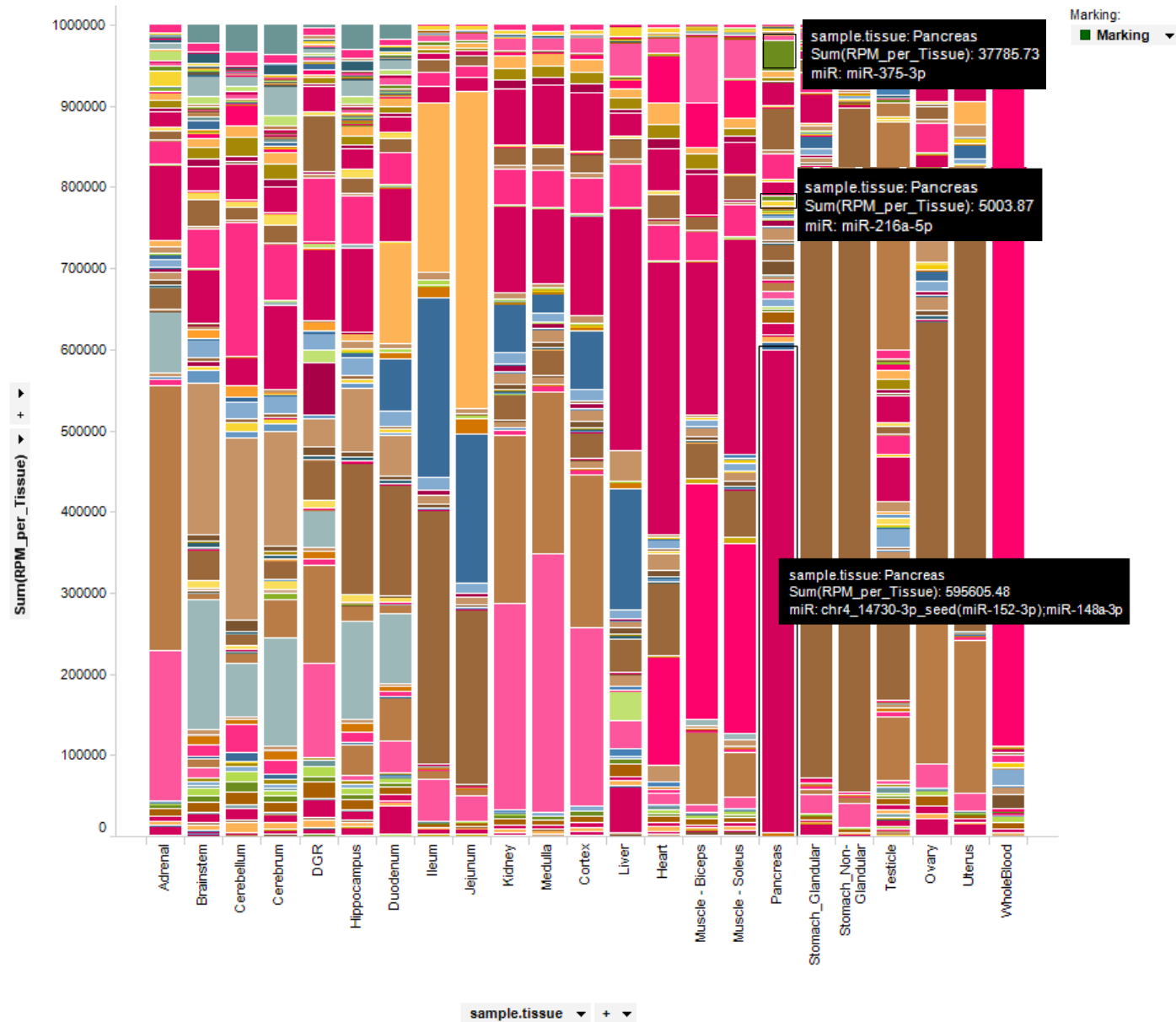

Supplement: Additional file 17: Figure S7. — Representation of rat miRNA sequencing data. Rat miRNA sequencing data is represented with the normalized reads per million on the Y-axis and the tissues on the X-axis. Each color within the figure represents an individual miRNA while the height of the colored bar represents the percentage of reads that a particular miRNA makes up within that tissue. MiRs-216a-5p, 375-3p and 148a-3p are indicated in the yellow, green and pink bars in the pancreas tissue respectively. (PDF 41 kb) [file 12864_2016_2956_MOESM17_ESM.pdf]

# Deep sequencing and qPCR inconsistency

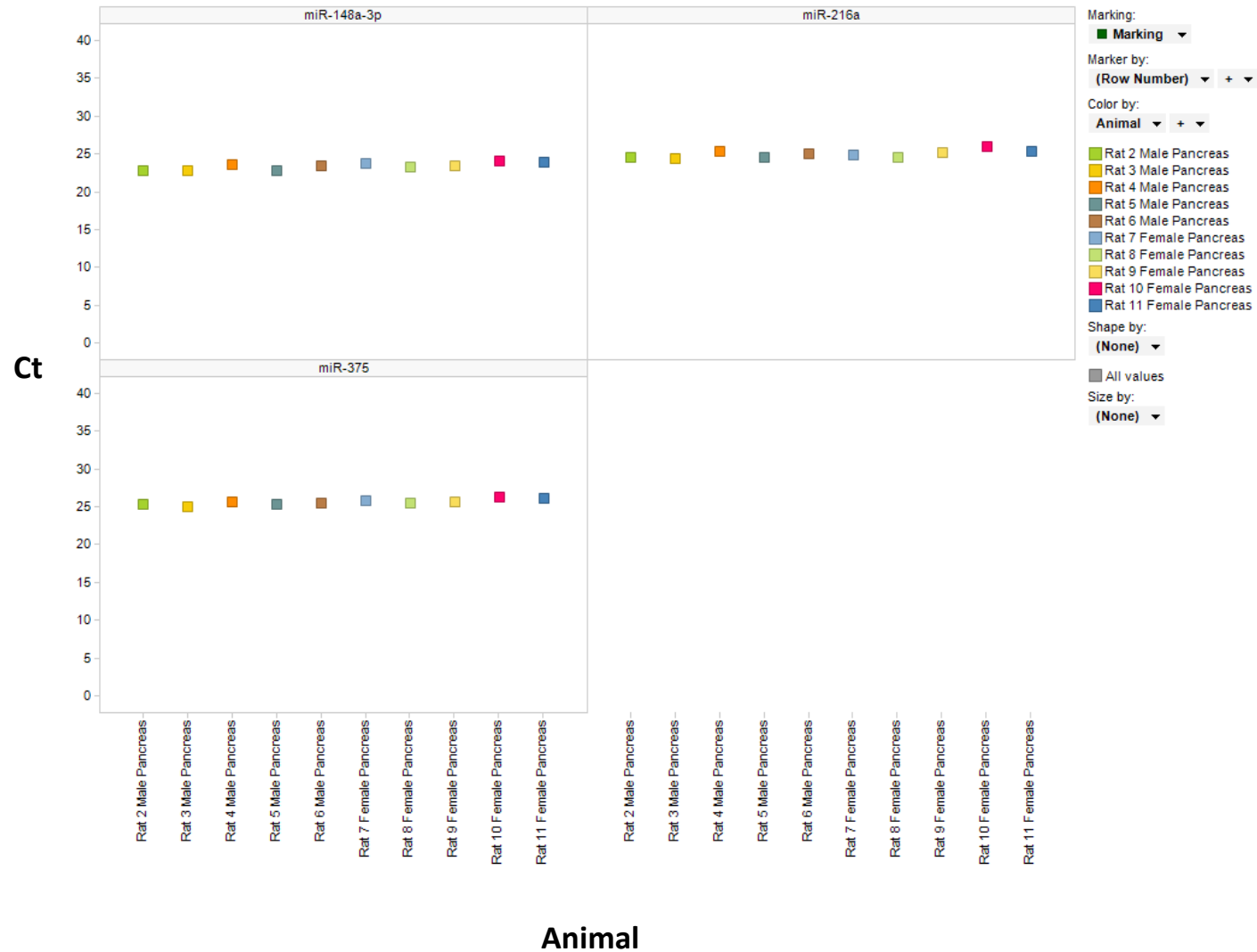

Supplement: Additional file 18: Figure S8. — qPCR of pancreas enriched miRNAs. Total RNA from the pancreas tissue of each individual rat was normalized by mass and examined by qPCR for expression of miRs-216a-5p, 375-3p and 148a-3p. Ct values are indicated on the Y-axis and the rat pancreas samples are indicated on the X-axis. Each miRNA appears to be expressed at approximately the same Ct value indicating they are present at roughly the same quantity in the pancreas. (PDF 32 kb) [file 12864_2016_2956_MOESM18_ESM.pdf]
